# Activation of IGF-1 pathway and suppression of atrophy related genes are involved in *Epimedium* extract (icariin) promoted C2C12 myotube hypertrophy

**DOI:** 10.1038/s41598-021-89039-0

**Published:** 2021-05-24

**Authors:** Yi-An Lin, Yan-Rong Li, Yi-Ching Chang, Mei-Chich Hsu, Szu-Tah Chen

**Affiliations:** 1 Department of Sports Medicine, Kaohsiung Medical University, Kaohsiung City, Taiwan; 2 Division of Endocrinology and Metabolism, Chang Gung Memorial Hospital, Chang Gung University, Taoyuan City, Taiwan; 3 Graduate Institute of Athletics and Coaching Science, National Taiwan Sport University, Taoyuan City, Taiwan

**Keywords:** Cell biology, Molecular biology, Physiology, Endocrinology

## Abstract

The regenerative effect of *Epimedium* and its major bioactive flavonoid icariin (ICA) have been documented in traditional medicine, but their effect on sarcopenia has not been evaluated. The aim of this study was to investigate the effects of *Epimedium* extract (EE) on skeletal muscle as represented by differentiated C2C12 cells. Here we demonstrated that EE and ICA stimulated C2C12 myotube hypertrophy by activating several, including IGF-1 signal pathways. C2C12 myotube hypertrophy was demonstrated by enlarged myotube and increased myosin heavy chains (MyHCs). In similar to IGF-1, EE/ICA activated key components of the IGF-1 signal pathway, including IGF-1 receptor. Pre-treatment with IGF-1 signal pathway specific inhibitors such as picropodophyllin, LY294002, and rapamycin attenuated EE induced myotube hypertrophy and MyHC isoform overexpression. In a different way, EE induced MHyC-S overexpression can be blocked by AMPK, but not by mTOR inhibitor. On the level of transcription, EE suppressed myostatin and MRF4 expression, but did not suppress atrogenes MAFbx and MuRF1 like IGF-1 did. Differential regulation of MyHC isoform and atrogenes is probably due to inequivalent AKT and AMPK phosphorylation induced by EE and IGF-1. These findings suggest that EE/ICA stimulates pathways partially overlapping with IGF-1 signaling pathway to promote myotube hypertrophy.

## Introduction

Skeletal muscle is the largest organ of human body responsible for movement, physical activity, energy metabolism, and myokine production^1^. Skeletal muscle atrophy and sarcopenia have been recognized to increase the risk of mortality in populations with chronic diseases, such as diabetes, heart failure, renal failure, and cancer^2,3^. In addition, ageing, physical inactivity/disuse, nutritional deficiency, excessive exercise and psychological stress also lead to a decline in muscle mass or strength^4–6^. In contrast, muscle hypertrophy is defined as an increase in muscle size or protein content via fusion of satellite cells and positive protein balance to pre-existing muscle fibers through several mechanisms^7^. Degradation and transition of myosin heavy chain (MyHC) isoforms, the major components of muscle cells, have been extensively investigated in various atrophic models such as dexamethasone abuse, oxidative stress, inflammation, denervation, and ageing^8,9^.

Various pathways have been demonstrated to regulate muscle cell growth^10,11^, among which, the locally derived insulin-like growth factor-1 (IGF-1) is especially important due to its simultaneously promoting and inhibiting protein synthesis and degradation^12–14^. Two important signal transduction pathways i.e. AKT/mammalian target of rapamycin (mTOR)/ P70S6K and mitogen-activated protein kinases (MAPK)/extracellular signal-regulated kinases (ERK) pathways^7,11^ were activated by IGF-1 and insulin. Activation of these pathways contribute to myogenesis not only by mediating the expression of myogenesis regulatory factors (MRFs) but also by enhancing dynamic protein balance^14–16^. Meanwhile, activation of the IGF-1 pathway ameliorates proteolysis induced by the ubiquitin–proteasome system^12,14^, and another atrophic signaling, the myostatin (MSTN)/Smad pathway^17,18^. Additionally, through diverse but redundant pathways, anabolic steroids such as testosterone synergistically liaise with growth factors (such as IGF-1) to mediate muscle development and maintenance. The androgenic effect was conducted through the binding of testosterone to androgen receptor (AR)^19,20^.

MSTN, a member of the transforming growth factor-β superfamily, serves as an IGF-1 counter-regulator and a skeletal muscle growth inhibitor via its activation of Smad transcription factors. Ample of evidence have shown that downregulation of MSTN not only prevents muscle atrophy but also induces muscle hypertrophy^17,21,22^. How MSTN regulates muscle mass is complex and redundant; for instance, crosstalk exists between IGF-1 and MSTN pathways, e.g. activation of AKT suppressed MSTN effect to inhibit myoblast differentiation^18,23^. In contrast, inactivation of AKT by dexamethasone or LY294002 resulted in an increase in MSTN mRNA via the activation of cyclic adenosine monophosphate (cAMP) response element-binding protein^24^; alternatively, it has been proved that IGF-1may directly suppress MSTN expression^22^. Meanwhile, follistatin, a specific MSTN inhibitor, requires the activation of IGF-1/AKT signal to retain its hypertrophic effects^25,26^.

The genus *Epimedium* Linn. (family Berberidaceae), also called yin yang huo or xianlinpi, is widely used in traditional Chinese medicine to improve kidney, osteoporosis, rheumatism, and sexual dysfunction^27^. Various flavonoids including epimedin A, B, C, and icariin (ICA) derived from *Epimedium* species were considered as main potential components^28^. ICA, as the crucial bioactive compound of *Epimedium* extract (EE), has been widely proved and applied in many studies of its protective effects on osteoporosis, never injury, and myocardial ischemia–reperfusion damage^29^. Moreover, *Epimedium* is considered to be a common Yang-invigorating Chinese herbal for improving energy production and muscular power or sexual function in males. In rodent studies, EE and ICA have been shown to have an androgenic-boosting effect by increasing testosterone levels^30,31^.

Our previous study of the attenuated serum creatine kinase activity post forced swimming^32^ suggested that EE treatment protects muscle strength through the promotion of muscle growth and function. Although the mechanism of EE/ICA remained obscured, a recent study showing that icaritin, a major intestinal metabolite of ICA, activated the phosphatidylinositol 3 kinase (PI3K)/AKT/FoxO signaling pathway in C2C12 myoblasts^33^, in along with that ICA treatment activated insulin receptor substrate-1 (IRS-1) and AMP-activated protein kinase (AMPK) signal pathways to improve insulin resistance in skeletal muscle cells^34^ suggest that the IGF-1/AKT pathway activation may play a critical role in the aforementioned effects of EE/ICA. Albeit potential benefits of EE and ICA for various diseases have been extensively studied, the therapeutic effects of EE and ICA on myogenesis and muscle hypertrophy are still lacking. Therefore, we conducted this study to evaluate the hypothesis that EE and its bioactive compound ICA enhanced muscle cell hypertrophy by the IGF-1 signaling pathway in differentiated C2C12 cells.

## Results

### EE promotes C2C12 cells hypertrophy and MyHC overexpression

**Figure 1:**
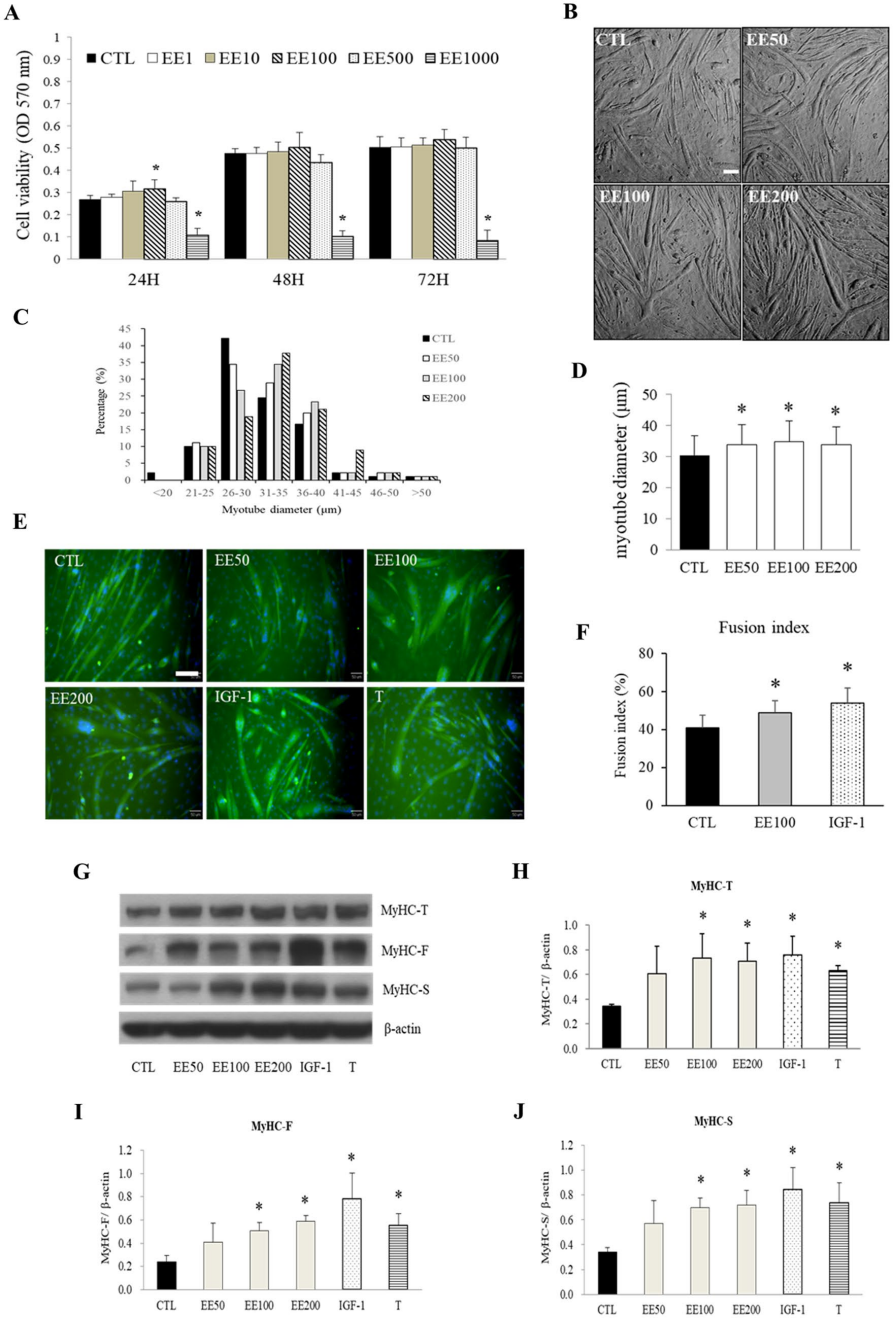
EE promoted C2C12 myotube hypertrophy and MyHC expression. **(A)** MTT assay was applied to determine the viability of undifferentiated C2C12 cells under various concentrations of EE at 24, 48, or 72 h. **(B)** Differentiated C2C12 cells were incubated in serum-free DMEM supplemented with 0 (CTL), 50, 100, or 200 μg/ml of EE. Representative photos were taken by a light microscope (scale bar, 100 μm) 48-h after EE treatment, which was then applied for measurement of myotube diameter (N = 90/group, data from three independent experiments). **(C)** The majority of C2C12 myotbe was measured around 26 to 30 μm under normal culture condition, various concentration of EE treatment significantly increased the average myotube diameter around 31 to 35 μm. **(D)** After 48-h treatment of vehicle (CTL), EE (50, 100, 200 μg/ml), IGF-1 (20 ng/ml), or testosterone (T, 500 nM), cells were stained with an immunofluorescent antibody against MyHC-T (green) and DAPI (blue). Scale bar, 100 μm. **(E)** Fusion index analysis indicated the percentage of nuclei in MyHC-positive myotube was increased after 100 μg/ml EE and IGF-1 treatment (N = 12 fields/group). **(F)** The abundance of MyHC expressed in C2C12 cells after 24-h treatment with vehicle (CTL), EE (50, 100, 200 μg/ml), IGF-1 (20 ng/ml), or testosterone (T, 500 nM) was evaluated by western blot assay (N = 4–6). The volume of bands stood for MyHC isoforms were digitally scanned and quantified by densitometry. After standardized with β-actin, the calculated densitometry data indicated that 24-h EE (100 and 200 μg/ml) treatment significantly increased the expression levels of MyHC-T (total, **G**), MyHC-F (fast type, **H**), and MyHC-S (slow type, **I**). IGF-1 and testosterone treatment served as positive controls. All data were expressed as mean ± SD. The symbol * stands for p < 0.05 as compared to CTL.

At the late stage of differentiation, C2C12 cells present not only abundant muscle-specific genes but also possess various physiological functions (e.g. muscle contraction and myokine secretion), which is more closed to real muscle fiber in vivo and suitable to be applied for investigating muscle maintenance^35–37^. By using thiazolyl blue tetrazolium bromide (MTT) assay, a cytotoxic effect of EE ranging from 1 to 1000 μg/ml were studied in undifferentiated C2C12 cells to determine the optimal effective and lethal doses. Our results showed that 100 μg/ml of EE slightly but significantly increased cell numbers at 24 h; whereas, 1000 μg/ml of EE brought to significant toxic effect at all time points (Fig. 1A). Base on this finding, concentrations of 50, 100, and 200 μg/ml of EE were tested to verify the hypertrophic effect of differentiated C2C12 cells. For comparison, IGF-1 (20 ng/ml) and testosterone (500 nM) were applied throughout this study as positive controls. As a result, 50–200 μg/ml of EE was found to significantly broaden myotube size (11.3, 11.8, and 10.4% by 50, 100, and 200 μg/ml of EE; respectively, by measuring myotube diameter, Fig. 1B–D). The fusion index of C2C12 cells was simultaneously increased by EE (8.01%) as it did by IGF-1 (13.5%) (Fig. 1E,F). In concomitant, the abundance of total MyHC (MyHC-T) protein, including the fast (MYH1/2/4, MyHC-F) and the slow (MYH7, MyHC-S) isoforms, was also increased by both 100 and 200 μg/ml of EE to the comparable levels induced by IGF-1 and testosterone (Fig. 1G–J).

### EE activates IGF-1 signal pathway

**Figure 2:**
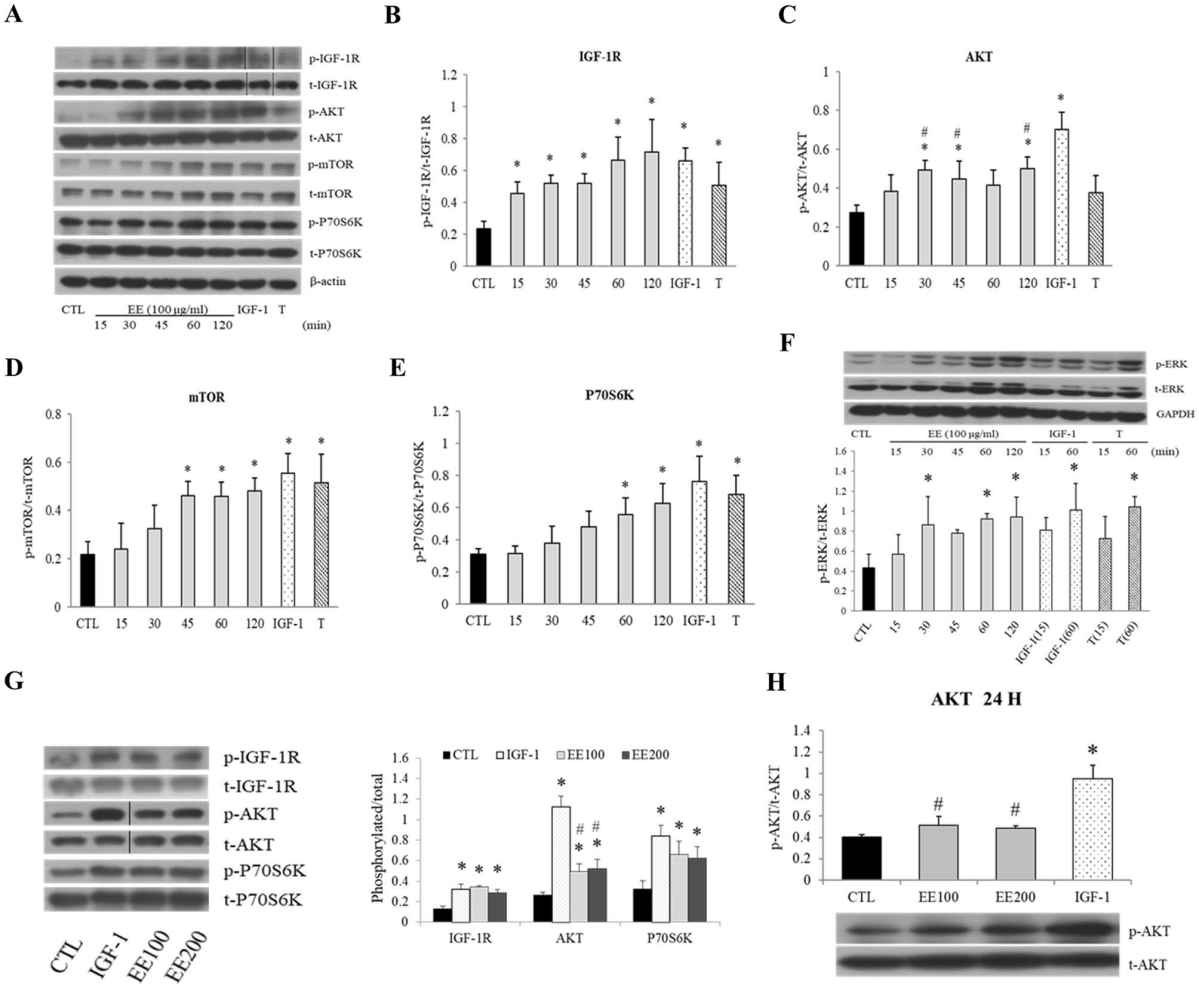
EE stimulated IGF-1 signal pathway in C2C12 cells. Differentiated C2C12 cells were overnight starved in serum-free medium before the indicated treatment. The levels of phosphorylated and total proteins before (CTL) and after EE (100 or 200 μg/ml) treatment were evaluated by Western blot for indicated time intervals (15–120 min). IGF-1 (20 ng/ml for 1 h) and testosterone (T, 500 nM for 1 h) treatment represented positive controls. **(A,F)** Western blot was performed by using specific antibodies against the phosphorylated and total proteins associated with the IGF-1 signal pathway. After standardized to β-actin or GAPDH, the level of target protein phosphorylation was semi-quantified by phosphorylated/total protein ratio, as shown in the followings IGF-1R **(B)**, AKT **(C)**, mTOR **(D)**, P70S6K **(E)**, and ERK **(F)** (N = 3–5). EE also activated all of the aforementioned proteins in a time sequence manner, only that the percentage of p-AKT was significantly lower than that induced by IGF-1 **(G)**. After 24 h treatment, the ratio of the IGF-1 induced p-AKT was still significantly higher than those induced by EE **(H)** (N = 4). All data were expressed as mean ± SD. The symbol * stands for p < 0.05 as compared to CTL; the symbol ^#^ stands for p < 0.05 as compared to IGF-1.

The effect of EE on IGF-1 signaling pathway was assessed in a time course assay. After an overnight serum-free starvation, EE (100 μg/ml) obviously enhanced IGF-1 receptor (IGF-1R) and its down-stream adaptor/effector protein phosphorylation in differentiated C2C12 cells in a time-dependent manner (Fig. 2A). EE induced IGF-1R and AKT phosphorylation since 15 min and plateaued at 30 min (Fig. 2A–C); the phosphorylation of mTOR (Fig. 2A,D) and P70S6K (Fig. 2A,E) were induced after 45 and 60 min of exposure, respectively. Meanwhile, EE also induced ERK phosphorylation at 30, 60, and 120 min (Fig. 2F). Notably, IGF-1 treatment showed a much rapid stimulation on IGF-1R and AKT phosphorylation at 5 min, and this was rather different from the relatively late phosphorylation of IGF-1R and AKT induced by EE and tyrosine-protein phosphatase (PTP1B) inhibitor (supplementary Fig. S1). In addition, it was interestingly to find that IGF-1 significantly induced heavier AKT phosphorylation than EE did from the beginning (Fig. 2G) and persisted throughout 24 h (Fig. 2H). In summary, EE induced similar phosphorylation patterns of AKT and mTOR, P70S6K, and ERK pathway to that induced by IGF-1 during a 2-h time course, albeit to a less extent in AKT phosphorylation.

### EE induced C2C12 myotube hypertrophy was blocked by inhibitors of IGF-1 signal pathway

**Figure 3:**
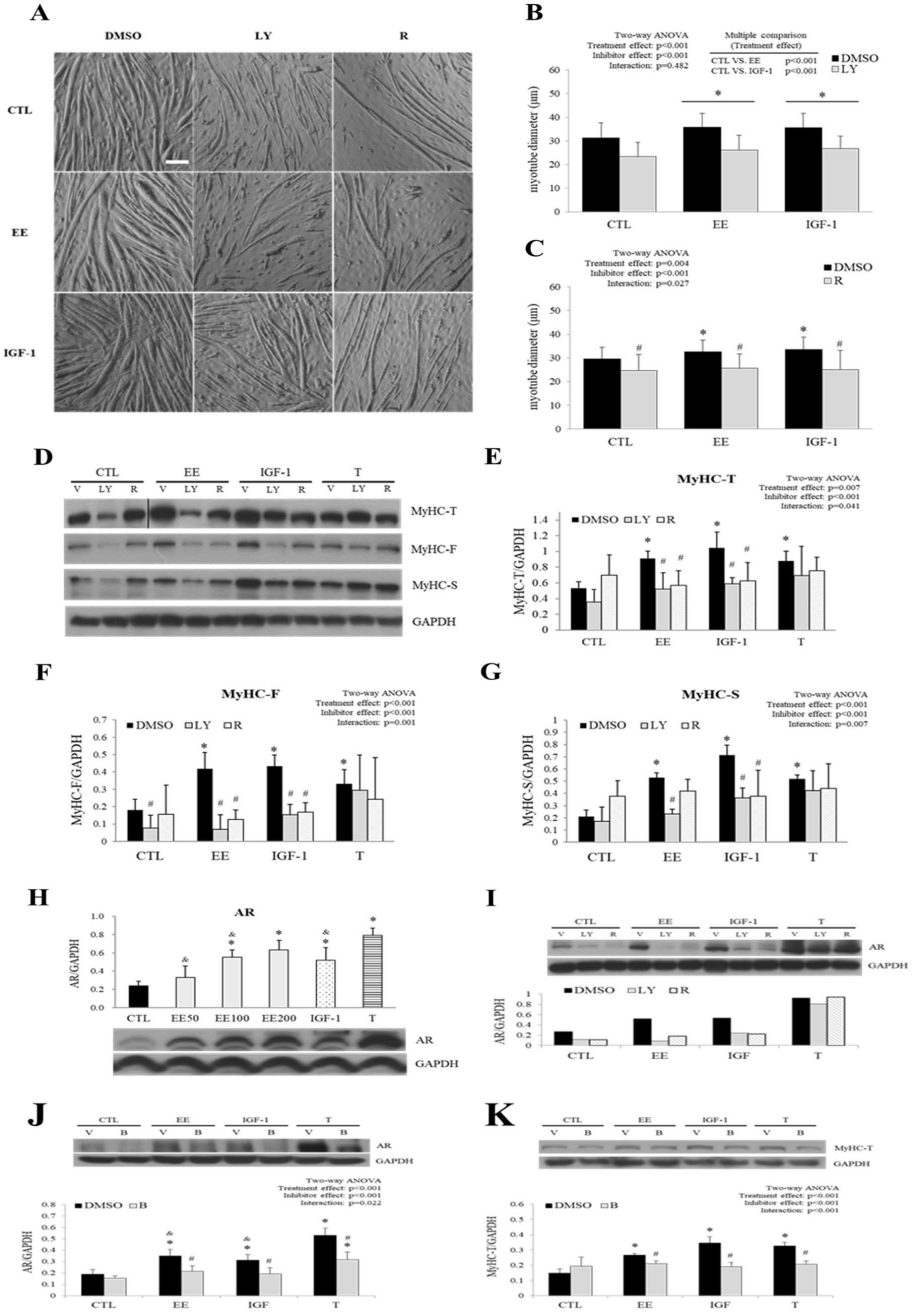
PI3K and mTOR inhibitors abolished EE-induced myotube hypertrophy. Specific inhibitors LY294002 (LY, 10 μM), rapamycin (R 20 nM), or bicalutamide (B, 20 μM) were applied 30 min before EE (100 μg/ml), IGF-1 (20 ng/ml), testosterone (T, 500 nM) or control (CTL) treatment. DMSO (0.1%) served as the vehicle control (V). Differentiated C2C12 cells were maintained in serum-free medium and pre-treated with inhibitors or DMSO followed by indicated treatment for 24 h (western blot) or 48 h further (morphological analysis). **(A)** Representative photos were taken at the end of the indicated treatment by a light microscope (scale bar, 100 μm). **(B,C)** Myotube hypertrophy was determined by measurement of myotube diameter (N = 75/group). **(D)** The abundance of MyHC [total (T) as well as fast (F) and slow (S) isoforms] in cells treated with indicated regimen was detected by western blot, and the quantitative results are shown in **(E–G)** (N = 3–6). **(H)** The abundance of AR after treated for 24 h with EE, IGF-1, and T was examined with western blot. The effects of IGF-I inhibitor and T antagonist on AR were also detected in **(I–K)** (N = 3–5). All data were expressed as mean ± SD. The symbol * stands for p < 0.05 as compared to the basal (CTL or CTL-DMSO); the symbol ^#^ stands for p < 0.05 as compared to the intra-group DMSO treatment; the symbol ^&^ stands for p < 0.05 as compared to T or T-DMSO treatment.

Inhibitors of IGF-1 signal pathway, such as LY294002 (PI3K inhibitor) and rapamycin (mTOR inhibitor), were applied 30 min prior to EE, IGF-1, and testosterone treatment to evaluate the influence of their effects on myotube hypertrophy and the expression levels of MyHC. We found that IGF-1 and EE induced myotube hypertrophy and MyHC isoforms up-regulation was inhibited significantly by LY294002 and rapamycin pre-treatment (Fig. 3A–G). However, testosterone induced MyHC isoforms overexpression was not repressed by neither inhibitor (Fig. 3D–G). Interestingly, while rapamycin blocked up-regulation of all of the IGF-1 induced MyHC isoforms (Fig. 3D–G), it could only abolish MyHC-T and MyHC-F, but not MyHC-S, up-regulation induced by EE (Fig. 3G). This differential response to rapamycin suggests that EE may also stimulate MyHC-S isoform expression through mTOR-independent pathway(s). Notably, despite that testosterone induced mTOR phosphorylation (Fig. 2A,D), neither LY294002 nor rapamycin could inhibit testosterone induced MyHC overexpression, indicating distinct pathways employed by testosterone.

Since testosterone was a potent inducer of myotube hypertrophy and used as an alternative control in this study, the abundance of AR in C2C12 cells was also studied after EE, IGF-1, and testosterone treatment. In consistence with the literature^38,39^, our results showed that IGF-1, EE (100 and 200 μg/ml), and testosterone significantly induced AR expression (Fig. 3H). Again, EE and IGF-1, but not testosterone, induced AR overexpression was abolished by LY294002 and rapamycin (Fig. 3I). Interestingly, bicalutamide (AR antagonist) inhibited not only testosterone, but also EE and IGF-1 induced AR and MyHC-T overexpression (Fig. 3J,K), indicating distinct but redundant pathways might be employed by EE, IGF-1 and testosterone to regulate myotube hypertrophy.

### EE and ICA stimulate AMPK phosphorylation

**Figure 4:**
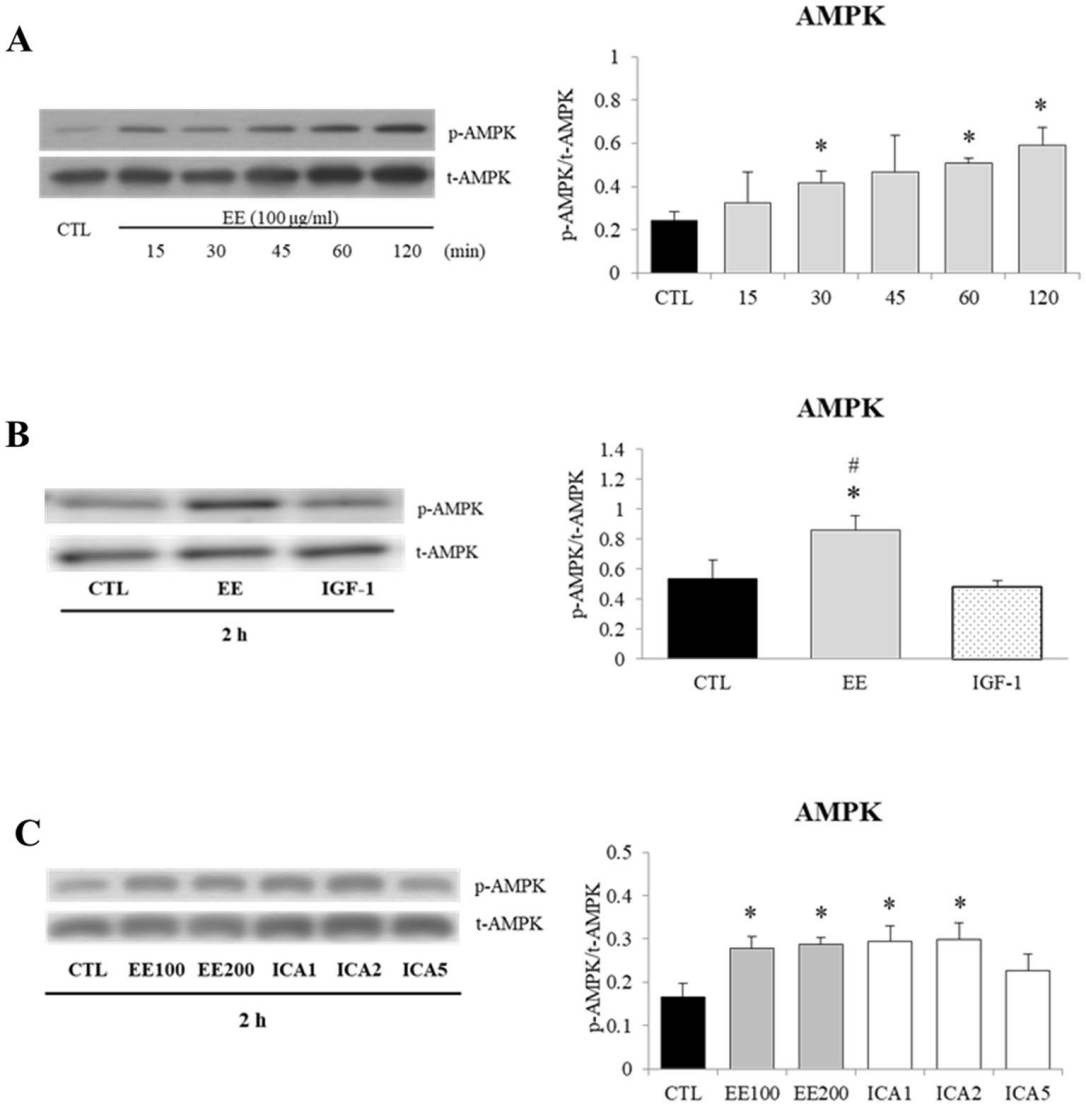
Effects of EE and ICA on AMPK/Thr172 phosphorylation. Differentiated C2C12 cells were starved in serum-free medium overnight before EE (100, 200 μg/ml), ICA (1–5 μM), and IGF-1 (20 ng/ml) treatment. Representative western blot images and corresponding densitometry measurements were shown and presented as phosphorylated/total protein ratios. **(A)** A time interval ranging from 15–120 min showed a sequential appearance of the phosphorylated AMPK induced by EE (100 μg/mL) (N = 5). **(B)** After a 2-h treatment, AMPK was activated by EE, but not by IGF-1 (N = 3). **(C)** ICA (1–2 μM) treatment induced a similar degree of AMPK phosphorylation as EE did (N = 3). All data were expressed as mean ± SD. The symbol * stands for p < 0.05 as compared to CTL, the symbol ^#^ represents p < 0.05 as compared to IGF-1.

Unlike the anabolic effect of IGF-1, activation of AMPK promotes catabolism such as energy production, fatty acid oxidation, mitochondria biogenesis, and protein degradation in skeletal muscle^40^. IGF-1 was reported to stimulate AMPK pathway through LKB1/ATM^41^, interestingly as shown in Fig. 4, EE induced even prominent phosphorylation of AMPK than IGF-1 did after 2-h incubation. As shown in Fig. 4C, ICA at the concentrations of 1 and 2 μM recapitulated AMPK phosphorylation induced by EE. Based on this finding, an AMPK inhibitor, compound C (CC), was included to examine whether the mTOR-independent regulation of MyHC-S by EE was AMPK mediated. As shown in supplementary Fig. S2, CC treatment slightly, but significantly, suppressed both EE and IGF-1 induced myotube hypertrophy; however, CC treatment could only suppress EE, but not IGF-1, induced MyHC-S overexpression, this may indicate that AMPK activation plays certain role in EE, but not IGF-1 induced MyHC-S expression.

### EE activated IGF-1 signal pathway through IGF-1R

**Figure 5:**
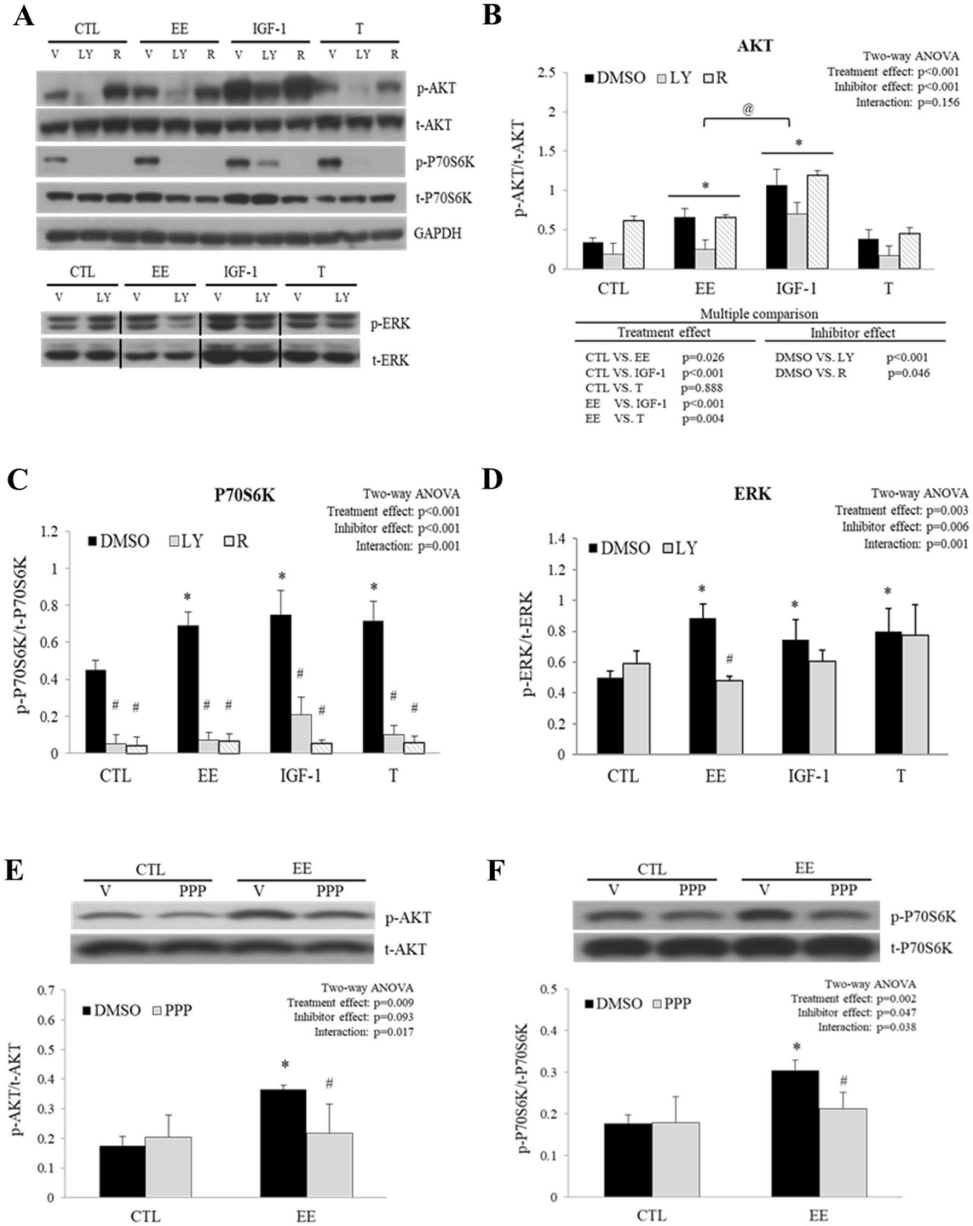
AKT and P70S6K phosphorylation induced by EE was attenuated by inhibitors targeted to the IGF-1 signal pathway. Differentiated C2C12 cells were starved in serum-free medium overnight before the treatment with control (CTL, no treatment), EE (100 μg/ml for 2 h), IGF-1 (20 ng/ml for 1 h), or testosterone (T, 500 nM for 1 h). In the reference group, cells were pre-treated with specific inhibitors such as LY294002 (LY, 10 μM), rapamycin (R, 20 nM), picropodophyllin (PPP, 5 μM), or 0.1% DMSO (served as vehicle control, V). The abundance of phosphorylated and total AKT, P70S6K, and ERK proteins was examined with western blots. Representative images are shown in **(A)** and the quantitative results are shown in **(B–D)** (N = 3–5). To prove that EE activated the cascade of IGF-1 signal pathway through IGF-1R, the selective IGF-1R antagonist PPP was included in the treatment. The representative AKT and P70S6K images and their quantitative ratios (phosphorylated/total) are shown in **(E,F)**, respectively (N = 4). All data were expressed as mean ± SD. The symbol * stands for p < 0.05 as compared to CTL or CTL-DMSO; the symbol ^#^ stands for p < 0.05 as compared to intra-group DMSO; and the symbol ^@^ stands for p < 0.05 as compared to IGF-1.

To examine the activation of key components of IGF-1 signal pathway, phosphorylation of AKT, P70S6K, and ERK was studied by western blot after EE, IGF-1, and testosterone treatment (Fig. 5A). We found that EE and IGF-1, but not testosterone, induced AKT phosphorylation. LY294002, but not rapamycin, significantly inhibited AKT phosphorylation induced by EE and IGF-1 (Fig. 5B). Pre-treatment with LY294002 and rapamycin abolished phosphorylated P70S6K induced by all of the regimens (Fig. 5C). EE induced ERK phosphorylation was abolished by LY294002, whereas IGF-1 and testosterone induced ERK phosphorylation were not suppressed (Fig. 5D), indicating that PI3K is required for EE induced ERK phosphorylation. To clarify whether EE activates IGF-1 signal pathway through IGF-1R, cells were pre-treated with an IGF-1R specific antagonist, picropodophyllin (PPP). As expexted, PPP attenuated EE induced AKT and P70S6K phosphorylation (Fig. 5E,F), MyHC-T overexpression and subsequent myotube hypertrophy (supplementary Fig. S3), suggesting that IGF-1R is involved in EE stimulated C2C12 myotube hypertrophy.

### ICA as the critical component of EE to promote myotube hypertrophy

**Figure 6:**
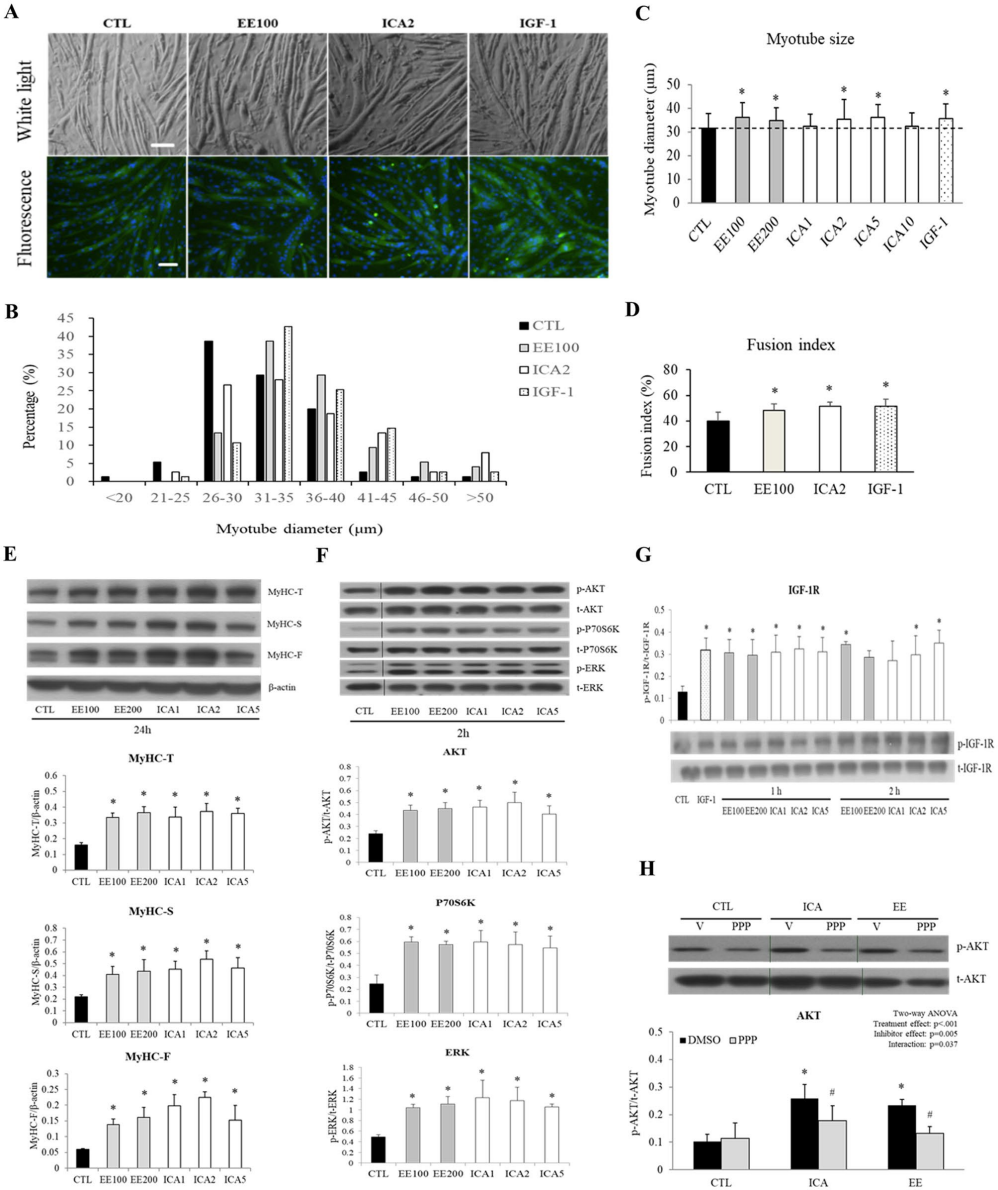
ICA equipotently induced myotube hypertrophy. Differentiated C2C12 cells were exposed to various concentrations of EE (100–200 μg/ml), ICA (1–5 μM), or IGF-1 (20 ng/ml) in serum-free medium for 1, 2, 24, or 48 h as indicated. **(A)** Representative images of bright fields (upper panel) and fluorescence MyHC (green) and DAPI (blue) of cells after 48-h incubation (scale bar, 100 μm). **(B,C)** Myotube diameter was measured by images captured from the light microscopy (N = 75/group). **(D)** Fusion index analysis indicated the percentage of nuclei in MyHC-positive myotube was increased after EE100, ICA2, and IGF-1 treatment (N = 12 fields/group). **(E–H)** Representative western blot images of MyHC isoforms and signaling transducers in cells treated with EE (100, 200 μg/ml) or ICA (1, 2, 5 μM) for the times indicated. Their quantitative results are shown at the lower panels (N = 3–4). In **(H)**, picropodophyllin (PPP, 5 μM) or 0.1% DMSO (served as vehicle control) was applied 2 h prior to CTL (basal), ICA (2 μM), or EE (100 μg/ml) treatment (N = 3–5). All data were expressed as mean ± SD. The symbol * stands for p < 0.05 as compared to CTL or CTL-DMSO; the symbol ^#^ stands for p < 0.05 as compared to intra-group DMSO.

Cells were treated with the equivalent molar ratio of purified ICA or EE to prove that the hypertrophic effect induced by EE was mediated by its component ICA (supplementary Fig. S3, each gram of dried EE powder contained 5.8 mg ICA). In the morphologic study, ICA at concentrations of 2–5 μM (ICA2 and ICA5) led to the significant increase in myotube diameter (11.5, 13.9, and 12.2% by ICA2, ICA5, and IGF-1, respectively) and fusion index (8.0, 11.3, and 11.1% by EE100, ICA2, and IGF-1, respectively) corresponding to the degree induced by EE and IGF-1 (Fig. 6A–D). Similar to EE, ICA at the range of 1 to 5 μM enhanced the abundance of all types of MyHC isoforms and peaked at 2 μM (Fig. 6E). As expected, ICA treatment remarkably enhanced IGF-1R, AKT, P70S6K, and ERK phosphorylation as EE did (Fig. 6F,G). Again, PPP pre-treatment abolished ICA induced AKT phosphorylation (Fig. 6H).

### Effects of EE on muscle-specific genes related to hypertrophy

**Figure 7:**
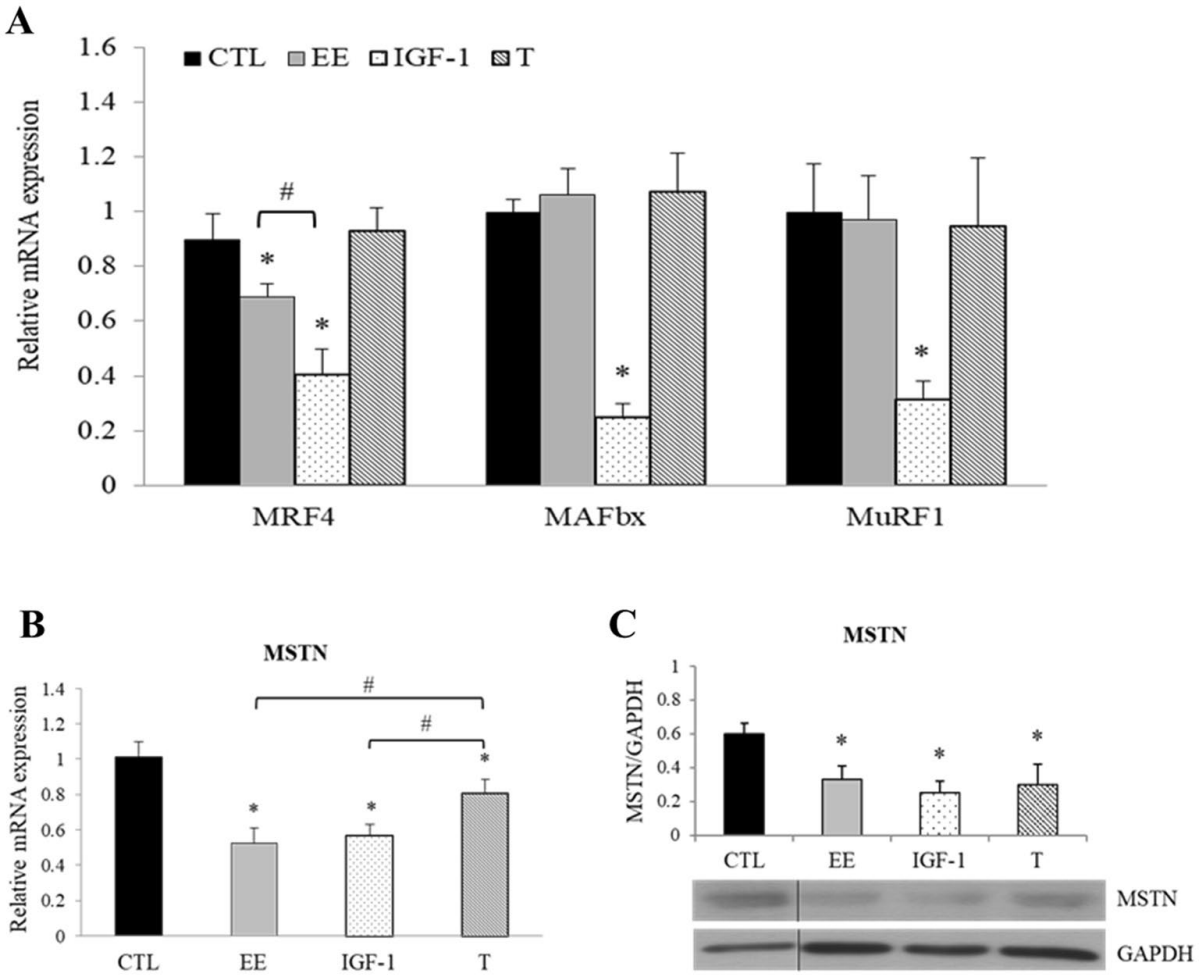
Muscle-specific genes related to hypertrophy regulated by EE. Differentiated C2C12 cells were treated with serum free medium (CTL), EE (100 μg/ml), IGF-1 (20 ng/ml), or testosterone (T, 500 nM). The change of indicated gene expression after 24 h treatment with aforementioned regimens was assessed by qPCR or western blot. **(A)** While IGF-1 significantly down-regulated the expression levels of MRF4, MAFbx and MuRF1 genes, EE could only down-regulate MRF4; and T has no regulatory effect (N = 5). **(B,C)** The expression of myostatin (MSTN; full-length precursor, 43 kDa) was down-regulated by IGF-1, EE and T (N = 4). The abundance of the PCR amplified fragment of the interested gene was measured and expressed as 2^−ΔΔCT^ after a correction to the simultaneously amplified GAPDH. All data were expressed as mean ± SD. The symbol * stands for p < 0.05 as compared to basal (CTL); the symbol ^#^ stands for p < 0.05 as compared to T.

The expression of muscle-specific genes related to hypertrophy (myogenic regulatory factor 4, MRF4) and atrophy [including muscle-specific ubiquitin E3-ligases muscle atrophy F-box (MAFbx or Atrogin-1), muscle RING finger 1 (MuRF1), and MSTN genes] before and after EE, IGF-1, or testosterone treatment was measured by quantitative reverse transcription-polymerase chain reaction (qRT-PCR). Despite a similar extent of morphological cellular hypertrophy induced by EE, IGF-1, and testosterone, the response of atrophy-associated muscle-specific genes to these treatments was quite different (Fig. 7A–C). IGF-1 significantly down-regulated MRF4, MAFbx, MuRF1, and MSTN genes, but testosterone could only suppress MSTN gene. Interestingly, although EE suppressed MSTN equivalently to the extent as IGF-1 did, it did not affect MAFbx and MuRF1 genes at all (Fig. 7). The suppression of MRF4 expression by EE and IGF-1 was surprising as it contradicts the hypertrophic effect. Taken together, IGF-1 is a much stronger suppressor of atrophic genes than EE and testosterone, and they might achieve this goal via partially overlapping but distinct pathways.

## Discussion

The present study demonstrates EE and its isolated compound ICA facilitate myotube hypertrophy in matured C2C12 cells through activating IGF-1 and AR signal pathways. A previous study suggested that icaritin promoted the phosphorylation levels of PI3K and its downstream protein/kinase in C2C12 myoblasts and counteracted muscle atrophy following mechanical unloading in mice^33^. This implies that the homologous compounds derived from ICA or ICA itself may possess the ability to improve myotube hypertrophy or prevent muscle atrophy. In this study, we further confirmed that EE supplement induced C2C12 cell hypertrophy in a dose-dependent manner (Fig. 1). By using western blot analysis, we found that EE and ICA induced C2C12 myotube hypertrophy associated with the increment of both fast (MyHC-F) and slow (MyHC-S) MyHC isoforms.

It has been reported that via as yet undefined mechanisms, EE and ICA improved cellular growth and regeneration through the activation of IGF-1 and insulin pathways in various cell lines^42–44^. In consistent with those findings, we found that EE and ICA activated IGF-1R and its downstream proteins/kinases, including AKT, mTOR, P70S6K, and ERK like IGF-1 did (Fig. 2). Interestingly, some lines of evidence also suggested that EE or ICA served as testosterone mimetics/stimulants to increase circulating testosterone and thus improve sexual function^30,45^. These observations suggest that EE or ICA may induce hypertrophic signals overlapping with IGF-1 and testosterone.

Specific inhibitors such as LY294002 (PI3K inhibitor) and rapamycin (mTOR inhibitor) were applied to demonstrate the involvement of IGF-1 pathway in EE induced C2C12 myotube hypertrophy. While LY294002 and rapamycin abolished IGF-1 induced myotube as it was expected, they also inhibited EE induced myotube hypertrophy to a similar extent (Fig. 3A–C). Noteworthy that while LY294002 and rapamycin down-regulated both IGF-1 and EE (but not testosterone) induced total MyHC (the sum of MyHC-F and -S isoforms), rapamycin could only suppress MyHC-F, but not MyHC-S isoform induced by EE (Fig. 3D–G), suggesting that EE may induce MyHC-S overexpression through mTOR-independent pathway(s). The distinct effect of mTOR regulation on MyHC isoform is also seen in the mTOR knockout study where selective reduction of fast-, but not slow-twitch muscles in a muscle-specific manner^46^.

A possible underlying mechanism is the over-activated AMPK induced by EE (Fig. 4), as increased MyHC-S but decreased MyHC-F expression has already been reported in C2C12 myotubes where AMPK is activated by various sources^47,48^. Moreover, this observation has been reported in the previous study showing ICA induced protein level and gene expression of irisin and peroxisome proliferator-activated receptor gamma co-activator 1 alpha (PGC-1α) in skeletal muscle via AMPK activation in vitro and in vivo^49^. This mechanism is further supported by the reversal of MyHC expression by AMPK inhibitor compound C seen in our study (supplementary Fig. S2) and by gene silencing reported in previous studies^50,51^. In addition to MyHC isoforms, the abundance of AR was also up-regulated by IGF-1^39^, EE, and testosterone. As the AR antagonist bicalutamide inhibited not only testosterone^52^, but also IGF-1 and EE induced AR and MyHC overexpression. This finding suggests that EE might also trigger AR to regulate MyHC expression, possibly through crosstalk with the IGF-1 pathway or other undefined pathways^39,53,54^.

In addition to AKT/P70S6K pathway, LY294002 pre-treatment also blocked EE-induced ERK phosphorylation. Most importantly, subsequent findings showed that the increment of AKT and P70S6K phosphorylation, MyHC-T protein level, and myotube diameter promoted by EE were diminished in the presence of IGF-1 antagonist, PPP (Fig. 5E,F; supplementary Fig. S3). These observations suggest that IGF-1 signaling pathway plays a critical role in EE regulated C2C12 cell myotube hypertrophy.

EE is an extract containing several bioactive flavonoids, among which, ICA is considered to be the major active component of EE (supplementary Fig. S4). To prove that it was the dominant functioning component, ICA, in equivalent concentrations were applied to evaluate the aforementioned effects of EE. As shown in Fig. 6, ICA at various concentrations (1–5 μM) induced comparable myotube hypertrophy, MyHC (both F- and S-types) overexpression, and phosphorylation of the IGF-1 pathway cascades such as IGF-1R, AKT, P70S6K, and ERK as EE did. Similarly, the phosphorylated AKT induced by ICA was also inhibited by PPP. Taken together, these data indicate that ICA should be the major bioactive component of EE promoting C2C12 myotube hypertrophy.

On the inspection of transcriptional level, it was unexpectedly to find that IGF-1 and EE significantly down-regulated MRF4 expression, since that down-regulation of MRF4 was reported in IGF-1 knockout mice^55^. However, a recent study found that knockout of MRF4 in adult muscle caused a striking increase in muscle size via activation of MEF2^56^. Since that MRF4 is the major MRF regulating postnatal skeletal muscle functions, more endeavor should be addressed to define its regulation by IGF-1 and EE signaling.

It has been known that myogenesis induced by IGF-1 results not only from up-regulation of anabolic MRFs but also from down-regulation of catabolic ubiquitin–proteasome^12,14^ and MSTN/Smad pathways^17,18^. Atrogenes MAFbx and MuRF1 are important E3 ubiquitin ligases directly regulated by the IGF-1/AKT/FoxO pathway, although IGF-1 and EE both activated the IGF-1/AKT/mTOR pathway, we found that only IGF-1 could suppress the expression of MAFbx and MuRF1. However, it has also been shown that co-incubation of AMPK enhancer, 5-aminoimidazole-4-carboxamide ribonucleotide (AICAR), abolished IGF-1 induced MAFbx and MuRF1 suppression in C2C12 myotubes^57^. Therefore, the finding of failure to suppress MAFbx and MuRF1 expression by EE could be attributed to the EE-induced AMPK activation (Fig. 4). This hypothesis is supported by a previous study showing that overexpression of a constitutively active AMPK mutant interfered with muscle anabolism^58^. Furthermore, AMPK can act as a critical repressor of protein synthesis through inhibiting mTOR complex 1 and promoting atrogenic activities^59^.

In agreement with previous studies^44,60^, our results indicate that IGF-1R/AKT/mTOR signaling plays an important role in EE/ICA-induced C2C12 myotube hypertrophy. However, a direct evidence suggesting ICA binds to and activates IGF-1R is lacking since that the phosphorylation of IGF-1R induced by EE/ICA was much weaker and slower than that induced by IGF-1. The discrepancy in effectors (AKT) phosphorylation and target genes (MyHC-S, MAFbx, and MuRF1) expression between EE/ICA and IGF-1 treatment may also hints for the application of different up-stream transducers by the two molecules. This line of evidence suggests that distinct but redundant signaling pathways are employed by EE/ICA and IGF-1 to trigger myotube hypertrophy. A possible regulatory mechanism was that EE and its major compound ICA may suppress phosphotyrosine phosphatase 1B (PTP1B) activity to modulate IGF-1/insulin signaling pathway^61,62^. However, it may not be appropriate to conclude that EE/ICA induced IGF-1R and downstream kinases activation by PTP1B inhibition, since that 2 μM of ICA is insufficient to suppress PTP1B activity (a 50% inhibition concentration, IC_50_, of ICA against PTP1B was > 300 μM^62^). Finally, an autocrine effect induced by EE/ICA can not be excluded since that increased IGF-1 mRNA level and peptide concentration has been reported in dermal papilla cells by ICA treatment^63^. Thus, although EE/ICA-induced myotube hypertrophy via the modulation of IGF-1R phosphorylation has been confirmed in this study, a direct IGF-1R interation or mediated through other underlying mechanisms require further clarification.

**Figure 8:**
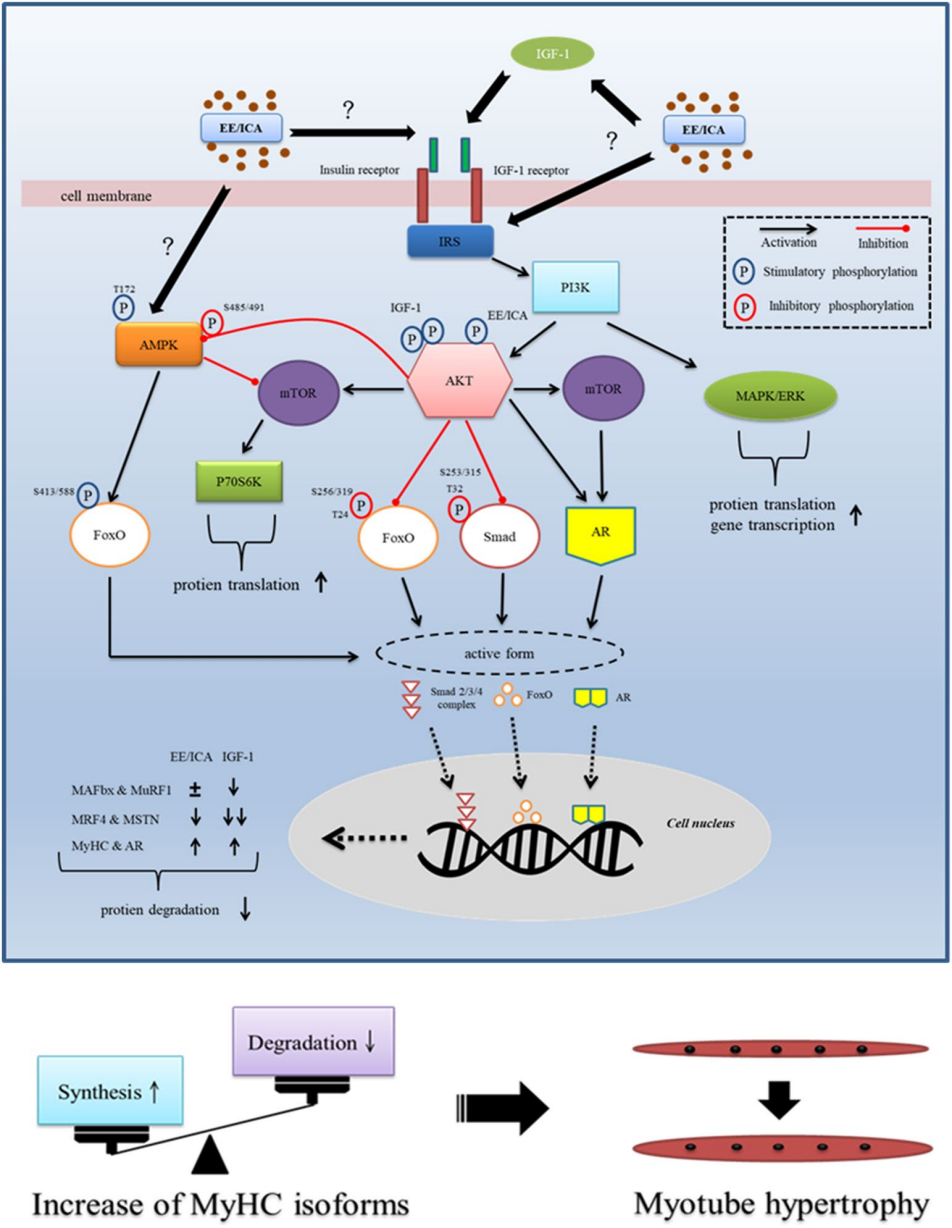
Schematic illustration showing EE and ICA promote myotube hypertrophy via IGF-1 signal pathway. Through triggering IGF-1R autophosphorylation, EE/ ICA activates key components of the IGF-1 signal cascades. As a consequence, the expression of MyHC and AR was up-regulated by the AKT/mTOR axis. In addition, two protein degradation signals, i.e. FoxO and MSTN/Smad are likely simultaneously inhibited by AKT to prevent from nuclear accumulation. Intranuclear FoxO induces atrogenes MAFbx and MuRF1 transcription, extranuclearly translocation of the phosphorylated FoxO induced by IGF-1 deactivates atrogene expression. Although EE and ICA may also reduce FoxO nuclear localization and MAFbx and MuRF1 transcription by activating AKT, the simultaneously activated AMPK/Thr172 reversely enhances FoxO function to result in a neutralizing effect on gene transcription. In contrast, heavier AKT phosphorylation induced by IGF-1 may induce AMPK/Ser485/491 phosphorylation instead, which may reduce the AMPK activity. The MSTN/Smad is another pathway to modulate protein degradation. The restricted function of Smad induced by AKT activation leads to a decrease of MSTN production. EE also suppresses the expression of MRF4 which is highly expressed in mature myotube as a growth repressor. To summarize, EE/ICA induces positive net protein balance by increasing MyHC isoforms and simultaneously suppressing atrogenes expression, which eventually leads to C2C12 myotube hypertrophy.

To summarize, regulatory mechanisms of EE- and/or ICA-induced myotube hypertrophy in differentiated C2C12 cells are illustrated as Fig. 8. Although the extent of myotube hypertrophy induced by IGF-1, EE, and testosterone was similar through the IGF-1 signal pathway, we found that IGF-1 synergistically suppressed MRF4, MAFbx, MuRF1, and MSTN; whereas testosterone could only suppress MSTN, and EE attenuated MRF4 and MSTN expression in differentiated C2C12 cells. To our knowledge, this is the first study reporting that EE has regulatory potential on the expression of MSTN and MRF4 at both mRNA and protein levels.

## Conclusion

In this study, we demonstrated that EE/ICA treatment enhanced myotube hypertrophy under serum-free status in differentiated C2C12 cells. Our results suggested that EE/ICA stimulated C2C12 myotube hypertrophy in conjunction with increased MyHC expression through several pathways, including AKT/mTOR, AMPK, and ERK pathways. As yet, EE attenuated MRF4 and MSTN, but not MAFbx and MuRF1 expression, which suggested that EE may protect muscle atrophy through MRF4 and MSTN modulation. This study indicates that EE and/or ICA may be applied as an alternative therapeutic strategy to improve growth factor deficit or metabolic impairment induced muscle wasting, such as sarcopenia.

## Methods

### Chemical reagents and EE

Chemicals such as MTT reagent, LY294002 (PI3K inhibitor), rapamycin (mTOR inhibitor), compound C (AMPK inhibitor), Long R^3^ IGF-1, testosterone, and icariin were purchased from Sigma-Aldrich (St. Louis, MO, USA). The IGF-1R specific inhibitor PPP was purchased from Selleck Chemicals (Houston, TX, USA). Bicalutamide, an AR antagonist, was purchased from ENZO Life Sciences (Farmingdale, NY, USA). The PTP1B inhibitor (PTP1B-IN-2) was purchased from MedChemExpress (Monmouth Junction, NJ, USA). EE powder purchased from the International Total Solution Taiwan Inc. (ITS-TW, Taipei, Taiwan) was dissolved (10 mg/ml, w/v) in phosphate-buffered saline (PBS) as a stock. In brief, raw *Epimedium* leaves (10 kg) were ground and extracted by boiled water for 3 times. The ratio of ground leaves to the solution was 1:10 (w/v). Finally, 1 kg EE powder (10 × concentrate) was collected and stored at room temperature after procedures of filtering, concentrating, and spray drying. The concentration of ICA in EE powder was determined by HPLC.

### Determination of ICA concentration in EE by HPLC

The concentration of ICA in EE was determined by HPLC–PDA method modified from Pharmacopoeia of The People's Republic of China^64^. Briefly, an HPLC system consisted of Waters 717plus autosampler (Waters, Milford, MA, USA), Waters 2996 photodiode array detector, Waters 600 Gradient delivery system, and Sugai U-620 Column heater (Sugai Chemie Inc.,Wakayama, Japan). Chromatographic separation of compounds was performed using a Cosmosil 5C18-MS-II (5 µm, 4.6 mm × 250 mm; Nacalai tesque, Kyoto, Japan) maintaining at 35℃. Elution was performed at a flow rate of 1 ml/min in a gradient. The mobile phase consisted of potassium dihydrogen phosphate aqueous solution, acetonitrile, and water. The UV detection wavelength was set at 270 nm. The peak of icariin eluted at approximately 44.6 min. Sample preparation was performed by adding 0.5 g of EE powder into 20 ml of methanol/water (70/30, v/v) for 35 min, then centrifuged at 3000 rpm for 10 min. The supernatant solution was collected and injected to HPLC for assay after filtered by a 0.45 µm PVDF filter. The results from HPLC chromatogram showed that each gram of dried EE powder contained 5.8 mg ICA (supplementary Fig. S4A,B).

### Cell culture

A murine skeletal myocyte cell line C2C12 was purchased from the Food Industry Research and Development Institute (Hsinchu, Taiwan). C2C12 cells were maintained at 37 °C in an atmosphere of 5% CO2. C2C12 myoblasts were cultured in growth medium (GM) composed of Dulbecco's modified Eagle's medium (DMEM; Biological Industries, Beit Haemek, Israel), 10% heat-inactivated fetal bovine serum (FBS), and 1% penicillin–streptomycin. For myotube differentiation, C2C12 cells were shifted to differentiation medium (DM) containing 2% horse serum to induce myotube formation on 80–90% confluency. Four days later, the differentiated C2C12 cells were maintained in serum-free medium overnight for indicated treatment.

### MTT assay

After a total of 7.5 × 10^3^ cells were seeded onto 24-well plate, the cytotoxic effects of EE on C2C12 myoblasts were assessed 24, 48, and 72 hs after incubation with various concentrations (1, 10, 100, 500, and 1000 μg/ml) of EE. In brief, cells were incubated with 200 μl/well of MTT reagent (0.5 mg/ml) at 37℃ for 2 h, the MTT metabolite, formazan, was then dissolved in 500 μl acidic isopropanol and detected by an ELISA reader under OD 570 nm.

### Morphological analysis: myotube diameter measurement and fusion index analysis

Cells were maintained in 60-mm culture dishes and allowed to differentiate for 4 days for morphological analysis. Differentiated C2C12 cells were applied to various treatments under a serum-free condition for 48 h. Myotube hypertrophy was assessed by myotube diameters measurement under 322.5 × magnitude by Olympus microscope CK30 (Olympus, Tokyo, Japan) and Dino-Eye Eyepiece digital camera with DinoCapture 2.0 software (AnMo Electronics Corporation, Hsinchu, Taiwan). A total of 15 microscopic fields were randomly captured from 3 independent experiments, and 5–6 intact myotubes were subsequently chosen from each field. The myotube diameter was eventually calculated as an average from 3 short-axis measurements along with the length of each myotube^65^. Meanwhile, immunofluorescence (IF) assay was used to investigate the degree of myotube hypertrophy. After washing 2 times with cold-PBS solution and fixation with 4% paraformaldehyde, cells were permeabilized with 0.3% Triton X-100 PBS solution, blocked with 0.1% Tween-20 PBS solution (PBST) containing 2% bovine serum albumin (BSA), and immunostained with total MyHC (MyHC-T) antibody (supplementary Table S2) overnight at 4℃. After washing with PBST solution, cells were incubated with Alexa488 antibody for 60 min at room temperature. Finally, culture dishes were mounted with mounting media containing nuclear dye 4′,6-diamidino-2-phenylindole (DAPI) after several washes in 0.1% PBST solution. Images were photographed by the Zeiss Axiovert 200 M Fluorescence Microscope with Axiovision software (Zeiss, Gottingen, Germany). The method of fusion index analysis was modified from the previous study and data was presented as the percentage of nuclei presented in myotubes to total nuclei in the captured field. A minimum of 3500 nuclei was counted from twelve random fields for each experimental condition^66^.

### Protein preparation and western blot

Cells were disrupted and harvested by scraping in RIPA lysis buffer after repetitive ice-cold PBS washing. After centrifuged at 4℃ for 15 min, supernatants containing whole cell lysates were collected. Protein concentration was measured by the Bradford protein assay (Bio-Rad, Hercules, CA, USA). Aliquots of 40 µg proteins were then fractionated on 6–12% SDS–polyacrylamide gels and transferred to PVDF membranes, which were then blocked with 0.1% Tween-20 tris-buffered saline (TBST) solution containing 5% nonfat milk or 5% BSA for 1 h at room temperature. Membranes were then overnight incubated with adequately diluted primary antibodies at 4℃. Antibodies against total/phosphorylated site of target proteins were listed in supplementary Table S1. Afterward, membranes were incubated with horseradish peroxidase-conjugated secondary antibodies (Merck Millipore) for 1 h at room temperature. The interested protein-antibody complexes were detected by enhanced Bio-Rad chemiluminescence substrate and visualized after exposure to X-ray films (Fuji, Kanagawa, Japan). The house-keeping genes GAPDH or β-actin were served as internal controls to assure equivalent loading of proteins in each immunoblot analysis. Once the abundance of the phosphorylated proteins has been determined, the abundance of the total proteins was detected on the same PVDF membrane after stripping. To determine the quantitative change, the volume of bands stood for interested proteins were digitally scanned and quantified by densitometry using the 1Dscan EX 3.0 software (Scanalytics Inc., Milwaukee, WI, USA).

### RNA preparation and reverse transcription-polymerase chain reaction (RT-PCR)

Total RNA was extracted with TRIzol reagent (Thermo Fisher, Waltham, MA, USA). In brief, cells were lysed in an adequate volume of TRIzol reagent and chloroform according to the instructions of the manufacturer. Total RNA was purified and stored at −80℃ until use. The concentrations of RNA were determined by a nanodrop spectrophotometer ND-1000 (Thermo Fisher). Total RNA was reverse-transcribed to single strand cDNA by the first strand cDNA synthesis kit (Genestar Biotech, Kaohsiung, Taiwan). The abundance of the interested gene expression was determined by using the Tools 2 × SYBR qPCR Mix (Biotools, New Taipei city, Taiwan) with the ABI 7900HT FAST real-time PCR system (Thermo Fisher) according to the manufacturer’s instruction. The abundance of the expression level of each interested gene was normalized to the endogenous control gene GAPDH and expressed as 2^−ΔΔCT^. Primers used for quantitative real-time PCR in this study were listed in supplementary Table S2.

### Statistical analysis

All data were expressed as mean ± SD. Statistical differences among groups were analyzed by one-way or two-way ANOVA with Tukey HSD or Dunnett T3 post hoc by using the IBM SPSS 20.0 software, and values of *p* < 0.05 were considered significant.

## Supplementary Information

Supplementary Information.

## Data Availability

All data generated and analyzed during this study are included in this published article and its Supplementary Information.

## References

[1] Schnyder S, Handschin C (2015). Skeletal muscle as an endocrine organ : PGC-1 α, myokines and exercise. Bone.

[2] Little JP, Phillips SM (2009). Resistance exercise and nutrition to counteract muscle wasting. Appl. Physiol. Nutr. Metab..

[3] Cohen S, Nathan JA, Goldberg AL (2015). Muscle wasting in disease: Molecular mechanisms and promising therapies. Nat. Rev. Drug Discov..

[4] Magne H, Savary-Auzeloux I, Rémond D, Dardevet D (2013). Nutritional strategies to counteract muscle atrophy caused by disuse and to improve recovery. Nutr. Res. Rev..

[5] Engelbrecht AM (2010). Daily brief restraint stress alters signaling pathways and induces atrophy and apoptosis in rat skeletal muscle. Stress.

[6] Alves Souza, R. W. *et al.* Resistance training with excessive training load and insufficient recovery alters skeletal muscle mass-related protein expression. *J. Strength Cond. Res.* **28**, 2338–2345. 10.1519/JSC.0000000000000421 (2014). 10.1519/JSC.0000000000000421 24531430

[7] Schiaffino S, Dyar KA, Ciciliot S, Blaauw B, Sandri M (2013). Mechanisms regulating skeletal muscle growth and atrophy. FEBS J..

[8] Holecek M (2012). Muscle wasting in animal models of severe illness. Int. J. Exp. Pathol..

[9] Romanick, M., Thompson, L.V & Brown-Borg, H. M. Murine models of atrophy, cachexia, and sarcopenia in skeletal muscle. *Biochim. Biophys. Acta* **1832**, 1410–20. 10.1016/j.bbadis.2013.03.011 (2013). 10.1016/j.bbadis.2013.03.011 PMC3687011 23523469

[10] Glass DJ (2005). Skeletal muscle hypertrophy and atrophy signaling pathways. Int. J. Biochem. Cell Biol..

[11] Knight JDR, Kothary R (2011). The myogenic kinome: Protein kinases critical to mammalian skeletal myogenesis. Skelet. Muscle.

[12] Stitt TN (2004). The IGF-1/PI3K/Akt pathway prevents expression of muscle atrophy-induced ubiquitin ligases by inhibiting FOXO transcription factors. Mol. Cell.

[13] Zanou N, Gailly P (2013). Skeletal muscle hypertrophy and regeneration: Interplay between the myogenic regulatory factors (MRFs) and insulin-like growth factors (IGFs) pathways. Cell. Mol. Life Sci..

[14] Sacheck JM, Ohtsuka A, McLary SC, Goldberg AL (2004). IGF-I stimulates muscle growth by suppressing protein breakdown and expression of atrophy-related ubiquitin ligases, atrogin-1 and MuRF1. Am. J. Physiol. Metab..

[15] Gao H, Ao M, Wang H, Yu L (2014). Rapamycin represses myotube hypertrophy and preserves viability of C2C12 cells during myogenesis in vitro. Transplantation.

[16] Haddad F, Adams GR (2004). Inhibition of MAP/ERK kinase prevents IGF-I-induced hypertrophy in rat muscles. J. Appl. Physiol..

[17] Rodriguez J (2014). Myostatin and the skeletal muscle atrophy and hypertrophy signaling pathways. Cell. Mol. Life Sci..

[18] Sartori R (2009). Smad2 and 3 transcription factors control muscle mass in adulthood. Am. J. Physiol. Physiol..

[19] Schiaffino S, Mammucari C (2011). Regulation of skeletal muscle growth by the IGF1-Akt/PKB pathway: Insights from genetic models. Skelet. Muscle.

[20] Hughes DC (2016). Testosterone enables growth and hypertrophy in fusion impaired myoblasts that display myotube atrophy: Deciphering the role of androgen and IGF-I receptors. Biogerontology.

[21] Mendler L, Baka Z, Kovács-Simon A, Dux L (2007). Androgens negatively regulate myostatin expression in an androgen-dependent skeletal muscle. Biochem. Biophys. Res. Commun..

[22] Hennebry A (2017). IGF1 stimulates greater muscle hypertrophy in the absence of myostatin in male mice. J. Endocrinol..

[23] Retamales A (2015). Insulin-like growth factor-1 suppresses the Myostatin signaling pathway during myogenic differentiation. Biochem. Biophys. Res. Commun..

[24] Xie Y, Perry BD, Espinoza D, Zhang P, Price SR (2018). Glucocorticoid-induced CREB activation and myostatin expression in C2C12 myotubes involves phosphodiesterase-3/4 signaling. Biochem. Biophys. Res. Commun..

[25] Zheng H (2017). Follistatin N terminus differentially regulates muscle size and fat in vivo. Exp. Mol. Med..

[26] Kalista S (2012). The type 1 insulin-like growth factor receptor (IGF-IR) pathway is mandatory for the follistatin-induced skeletal muscle hypertrophy. Endocrinology.

[27] Ma H (2011). The genus Epimedium: An ethnopharmacological and phytochemical review. J. Ethnopharmacol..

[28] Chen XJ (2007). Simultaneous determination of 15 flavonoids in Epimedium using pressurized liquid extraction and high-performance liquid chromatography. J. Chromatogr. A.

[29] Li C, Li Q, Mei Q, Lu T (2015). Pharmacological effects and pharmacokinetic properties of icariin, the major bioactive component in Herba Epimedii. Life Sci..

[30] Zhang ZB, Yang QT (2006). The testosterone mimetic properties of icariin. Asian J. Androl..

[31] Chen, M., Hao, J., Yang, Q. & Li, G. Effects of icariin on reproductive functions in male rats. *Molecules* **19**, 9502–9514. 10.3390/molecules19079502 (2014). 10.3390/molecules19079502 PMC6271987 24995929

[32] Lin YA, Khamoui AV, Liao CC, Huang CC, Hsu MC (2015). Improvement of exercise performance and attenuation of a marker of muscle damage by Epimedium Brevicornum supplementation in mice. Adapt. Med..

[33] Zhang ZK (2016). Icaritin requires Phosphatidylinositol 3 kinase (PI3K)/Akt signaling to counteract skeletal muscle atrophy following mechanical unloading. Sci. Rep..

[34] Han Y, Jung HW, Park YK (2015). Effects of Icariin on insulin resistance via the activation of AMPK pathway in C2C12 mouse muscle cells. Eur. J. Pharmacol..

[35] Brown DM, Parr T, Brameld JM (2012). Myosin heavy chain mRNA isoforms are expressed in two distinct cohorts during C2C12 myogenesis. J. Muscle Res. Cell Motil..

[36] Peake JM, Gatta PD, Suzuki K, Nieman DC (2015). Cytokine expression and secretion by skeletal muscle cells: Regulatory mechanisms and exercise effects. Exerc. Immunol. Rev..

[37] Furuichi Y, Manabe Y, Takagi M, Aoki M, Fujii NL (2018). Evidence for acute contraction-induced myokine secretion by C2C12 myotubes. PLoS ONE.

[38] Serra C (2011). The role of GH and IGF-I in mediating anabolic effects of testosterone on androgen-responsive muscle. Endocrinology.

[39] Lee WJ (2009). Insulin-like growth factor-I-induced androgen receptor activation is mediated by the PI3K/Akt pathway in C2C12 skeletal muscle cells. Mol. Cells.

[40] Kjøbsted R (2018). AMPK in skeletal muscle function and metabolism. FASEB J..

[41] Suzuki A (2004). IGF-1 phosphorylates AMPK-α subunit in ATM-dependent and LKB1-independent manner. Biochem. Biophys. Res. Commun..

[42] Song YH (2016). Icariin attenuated oxidative stress induced-cardiac apoptosis by mitochondria protection and ERK activation. Biomed. Pharmacother..

[43] Zhang D (2015). Icariin prevents amyloid beta-induced apoptosis via the PI3K/akt pathway in PC-12 cells. Evidence-Based Complement. Altern. Med..

[44] Zhou L (2021). Icariin ameliorates estrogen-deficiency induced bone loss by enhancing IGF-I signaling via its crosstalk with non-genomic ERα signaling. Phytomedicine.

[45] Ding J (2018). Icariin improves the sexual function of male mice through the PI3K/AKT/eNOS/NO signalling pathway. Andrologia.

[46] Risson V (2009). Muscle inactivation of mTOR causes metabolic and dystrophin defects leading to severe myopathy. J. Cell Biol..

[47] Chen X (2018). Arginine promotes skeletal muscle fiber type transformation from fast-twitch to slow-twitch via Sirt1/AMPK pathway. J. Nutr. Biochem..

[48] Xu M (2020). Procyanidin B2 promotes skeletal slow-twitch myofiber gene expression through the AMPK signaling pathway in C2C12 myotubes. J. Agric. Food Chem..

[49] Chen SQ (2019). Icariin induces irisin/FNDC5 expression in C2C12 cells via the AMPK pathway. Biomed. Pharmacother..

[50] Chen X (2018). Arginine promotes slow myosin heavy chain expression via Akirin2 and the AMP-activated protein kinase signaling pathway in porcine skeletal muscle satellite cells. J. Agric. Food Chem..

[51] Chen X (2019). Ferulic acid regulates muscle fiber type formation through the Sirt1/AMPK signaling pathway. Food Funct..

[52] Wannenes F (2008). Androgen receptor expression during C2C12 skeletal muscle cell line differentiation. Mol. Cell. Endocrinol..

[53] Wu Y, Bauman WA, Blitzer RD, Cardozo C (2010). Testosterone-induced hypertrophy of L6 myoblasts is dependent upon Erk and mTOR. Biochem. Biophys. Res. Commun..

[54] Sculthorpe N (2012). Androgens affect myogenesis in vitro and increase local IGF-1 expression. Med. Sci. Sports Exerc..

[55] Miyake M (2007). Myostatin and MyoD family expression in skeletal muscle of IGF-1 knockout mice. Cell Biol. Int..

[56] Moretti I (2016). MRF4 negatively regulates adult skeletal muscle growth by repressing MEF2 activity. Nat. Commun..

[57] Tong JF, Yan X, Zhu MJ, Du M (2009). AMP-activated protein kinase enhances the expression of muscle-specific ubiquitin ligases despite its activation of IGF-1/Akt signaling in C2C12 myotubes. J. Cell. Biochem..

[58] Williamson DL, Bolster DR, Kimball SR, Jefferson LS (2006). Time course changes in signaling pathways and protein synthesis in C 2C12 myotubes following AMPK activation by AICAR. Am. J. Physiol. Endocrinol. Metab..

[59] Sanchez AMJ (2012). The role of AMP-activated protein kinase in the coordination of skeletal muscle turnover and energy homeostasis. Am. J. Physiol. Cell Physiol..

[60] Jiang, M. C., Chen, X. H., Zhao, X., Zhang, X. J. & Chen, W. F. Involvement of IGF-1 receptor signaling pathway in the neuroprotective effects of Icaritin against MPP(+)-induced toxicity in MES23.5 cells. *Eur. J. Pharmacol.* **786**, 53–59. 10.1016/j.ejphar.2016.05.031 (2016). 10.1016/j.ejphar.2016.05.031 27238975

[61] Luo, J. *et al.* CYC31, a natural bromophenol PTP1B inhibitor, activates insulin signaling and improves long chain-fatty acid oxidation in C2C12 myotubes. *Mar. Drugs* **18**, 10.3390/md18050267 (2020). 10.3390/md18050267 PMC7281472 32438641

[62] Kim, D. H., Jung, H. A., Sohn, H. S., Kim, J. W. & Choi, J. S. Potential of icariin metabolites from *Epimedium koreanum* Nakai as antidiabetic therapeutic agents. *Molecules* **22**, 10.3390/molecules22060986 (2017). 10.3390/molecules22060986 PMC6152727 28608833

[63] Su YS (2017). Icariin promotes mouse hair follicle growth by increasing insulin-like growth factor 1 expression in dermal papillary cells. Clin. Exp. Dermatol..

[64] Chinese Pharmacopoeia Commission. *Pharmacopoeia of the People’s Republic of China*. (Chinese Pharmacopoeia Commission, 2015).

[65] Yeh TS, Hsu CC, Yang SC, Hsu MC, Liu JF (2014). Angelica Sinensis promotes myotube hypertrophy through the PI3K/Akt/mTOR pathway. BMC Complement. Altern. Med..

[66] Isobe M, Lee S, Waguri S, Kametaka S (2018). Clathrin adaptor GGA1 modulates myogenesis of C2C12 myoblasts. PLoS ONE.

