# Supplementary material for: Activation of IGF-1 pathway and suppression of atrophy related genes are involved in Epimedium extract (icariin) promoted C2C12 myotube hypertrophy
Source: Sci Rep. 2021 May 24;11:10790. doi: 10.1038/s41598-021-89039-0 (PMC8144409; doi:10.1038/s41598-021-89039-0)

Supplementary information

# Activation of IGF-1 pathway and suppression of atrophy related genes are involved in *Epimedium* extract (icariin) promoted C2C12 myotube hypertrophy

Yi-An Lin[1,2,3], Yan-Rong Li[2], Yi-Ching Chang[2], Mei-Chich Hsu[1*], Szu-Tah Chen[2*]

[1] Department of Sports Medicine, Kaohsiung Medical University, Kaohsiung City, Taiwan

[2] Division of Endocrinology and Metabolism, Chang Gung Memorial Hospital, Chang Gung University, Taoyuan City, Taiwan

[3] Graduate Institute of Athletics and Coaching Science, National Taiwan Sport University, Taoyuan City, Taiwan

*corresponding author (e-mail: stc1105@cgmh.org.tw or meichich@gmail.com)

Supplementary Table

## Supplementary Table S1. The list of antibodies

| Antibodies | Company | Product # | Dilution WB (IF) | Host |
|---|---|---|---|---|
| p-AKT Ser473 | Cell signaling | 9271S | 1:1000 | Rabbit |
| AKT | Cell signaling | 9272S | 1:5000 | Rabbit |
| p-AMPKα Thr172 | Cell signaling | 2531S | 1:2000 | Rabbit |
| AMPKα | Cell signaling | 2532S | 1:500 | Rabbit |
| p-ERK1/2 Thr185/Tyr187 | Millipore | 04-797 | 1:2000 | Rabbit |
| ERK1/2 | Millipore | 05-1152 | 1:5000 | Mouse |
| p-mTOR Ser2448 | Cell signaling | 2971S | 1:3000 | Rabbit |
| mTOR | Cell signaling | 2972S | 1:5000 | Rabbit |
| p-IGF-1Rβ Tyr1135/1136 | Cell signaling | 3024S | 1:500 | Rabbit |
| IGF-1Rβ | Cell signaling | 3027S | 1:2000 | Rabbit |
| p-P70S6K Thr389 | Cell signaling | 9029S | 1:2000 | Rabbit |
| P70S6K | Cell signaling | 9202S | 1:5000 | Rabbit |
| AR | Santa Cruz | SC-816 | 1:500 | Rabbit |
| β-actin | Sigma Aldrich | A5441 | 1:10000 | Mouse |
| GAPDH | GeneTex | GTX100118 | 1:10000 | Rabbit |
| MSTN | GeneTex | GTX32624 | 1:1000 | Rabbit |
| MYH (MyHC-T) | Santa Cruz | SC-20641 | 1:1000 (1:200) | Rabbit |
| MYH1/2/4 (MyHC-F) | Santa Cruz | SC-58797 | 1:250 | Mouse |
| MYH7 (MyHC-S) | ABclonal | A7564 | 1:3000 | Rabbit |
| Goat anti-rabbit IgG | Millipore | AP307P | 1:5000 | Goat |
| Goat anti-mouse IgG | Millipore | AP124P | 1:5000 | Goat |
| Alexa Flour 488 Goat-anti mouse IgG | Jackson Lab | 111-545-003 | 1:5000 | Goat |

AR: androgen receptor; GAPDH: glyceraldehyde 3-phosphate dehydrogenase; MSTN: myostatin; MyHC-F: myosin heavy chain-fast isoform; MyHC-S: myosin heavy chain-slow isoform; MyHC-T: myosin heavy chain-total isoform

Supplementary Table

**Supplementary Table S2. PCR primer sequences used for gene expression**

| Gene | Forward primer (5’-3’) | Reverse primer (5’-3’) |
|---|---|---|
| MRF4 | GTGGCCAAGTGTTTCGGATC | AAAGGCGCTGAAGACTGCTG |
| MSTN | GTCCAGAGGGATGACAGCAG | GTCTTGACGGGTCTGAGATA |
| MAFbx | AAGGAAGATGAACGCTGTCA | ATTGCCTCCCAGATAAAGTATGT |
| MuRF1 | AGCCAAGACAATAGAGATGCCTACTTC | GGCCTTGAACTCATAGAGATCCAAC |
| GAPDH | TTCACCACCATGGAGAAGGC | GGCATGGACTGTGGTCATGA |

GAPDH: glyceraldehyde 3-phosphate dehydrogenase; MAFbx: muscle atrophy F-box; MRF4: myogenic regulatory factor 4; MSTN: myostatin; MuRF1: muscle RING finger 1;

Supplementary Figure

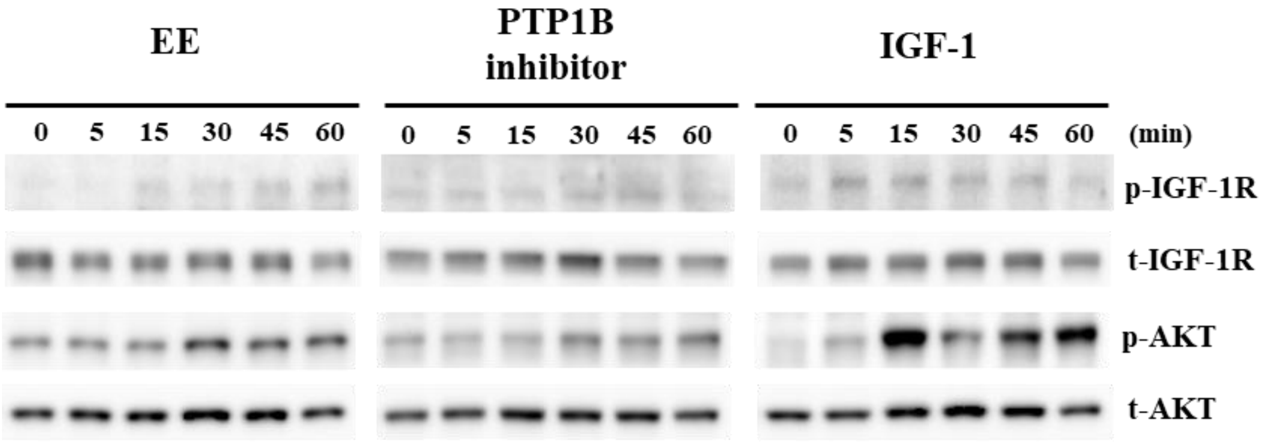


**Supplementary Figure S1. The time-dependent effect of EE, PTP1B inhibitor, and IGF-1 on IGF-1R and AKT phosphorylation.**

Differentiated C2C12 cells were starved overnight in serum-free medium before the indicated treatment. The levels of phosphorylated and total proteins before (0 min) and after treatments of EE (100 μg/ml), PTP1B inhibitor (10 μM), and IGF-1 (20 ng/ml) were evaluated by western blot for indicated time intervals (5-60 min) (N=3).

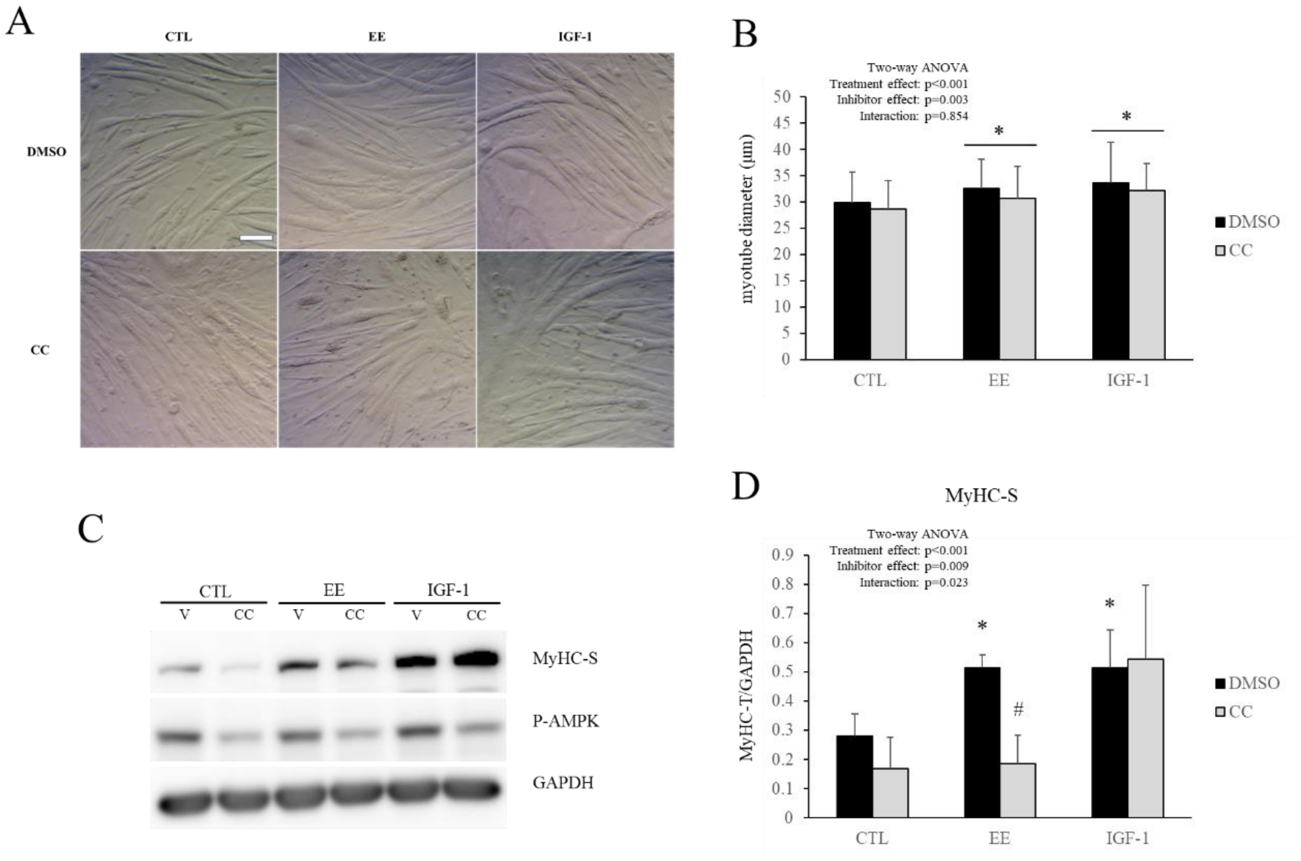


**Supplementary Figure S2. The effect of AMPK inhibition on EE and IGF-1 induced MyHC-S expression.**

AMPK inhibitor (compound C, CC; 10 μM) was applied 30 min before EE (100 μg/ml), IGF-1 (20 ng/ml) or control (CTL) treatment, where DMSO (0.1%) served as vehicle control (V). Differentiated C2C12 cells were maintained in serum-free medium overnight before pre-treated with CC or DMSO followed by indicated treatment for further 24h. (A) Representative images of differentiated C2C12 cells were taken at the end of the indicated treatment by a light microscope (scale bar, 100 μm). (B) The myotube diameter of each treatment group was determined by repetitive measurements of 90 myotubes in triplicate. Myotube hypertrophy was documented by the significant increment of myotube diameter as compared to CTL. Representative western blots (C) and densitometric analysis (D) showed both EE and IGF-1 induced AMPK phosphorylation and enhanced MyHC-S protein overexpression (N=3). CC treatment significantly abolished EE, but not IGF-1 induced MyHC-S overexpression. All data were expressed as mean ± SD. The symbol * stands for $p < 0.05$ as compared to the basal (CTL or CTL-DMSO); the symbol # stands for $p < 0.05$ as compared to the intra-group DMSO treatment.

Supplementary Figure

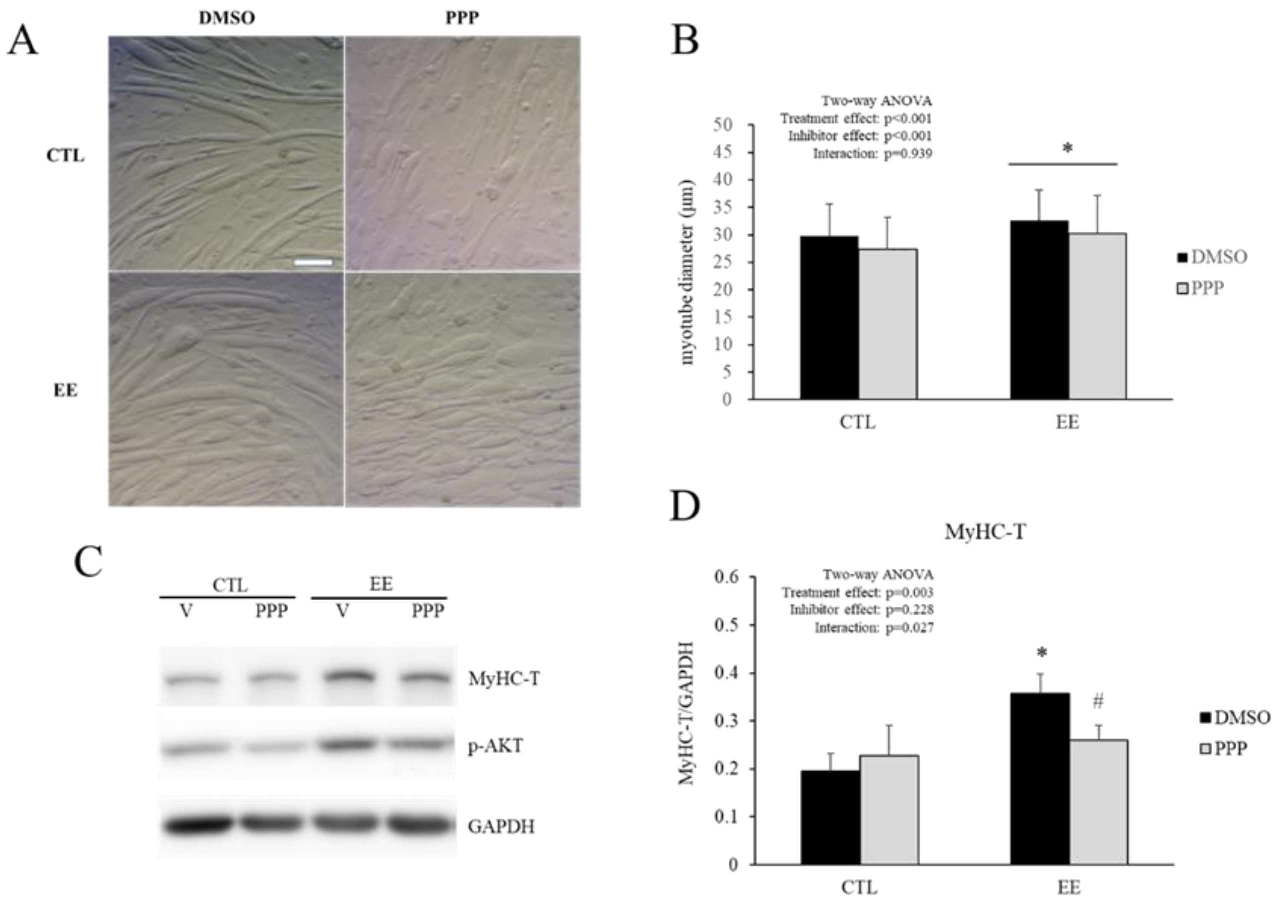


**Supplementary Figure S3. IGF-1R antagonist picropodophyllin blocked EE-induced myotube hypertrophy.**

Selective IGF-1R antagonist picropodophyllin (PPP, 5 μM) was applied 2h before EE (100 μg/ml) or control (CTL) treatment, where DMSO (0.1%) served as vehicle control (V). Differentiated C2C12 cells were maintained in serum-free medium overnight before pre-treated with PPP or DMSO followed by indicated treatment for further 24h. (A) Representative images of differentiated C2C12 cells were taken at the end of the indicated treatment by a light microscope (scale bar, 100 μm). (B) The myotube diameter of each treatment group was determined by repetitive measurements of 90 myotubes in triplicate. Myotube hypertrophy was documented by the significant increment of myotube diameter as compared to CTL. Representative western blots (C) and densitometric analysis (D) showed EE significantly induced AKT phosphorylation and MyHC-T overexpression (N=3). PPP suppressed EE induced myotube hypertrophy and MyHC-T overexpression. All data were expressed as mean ± SD. The symbol * stands for $p < 0.05$ as compared to the basal (CTL or CTL-DMSO); the symbol $^{\#}$ stands for $p < 0.05$ as compared to the intra-group DMSO treatment.

Supplementary Figure

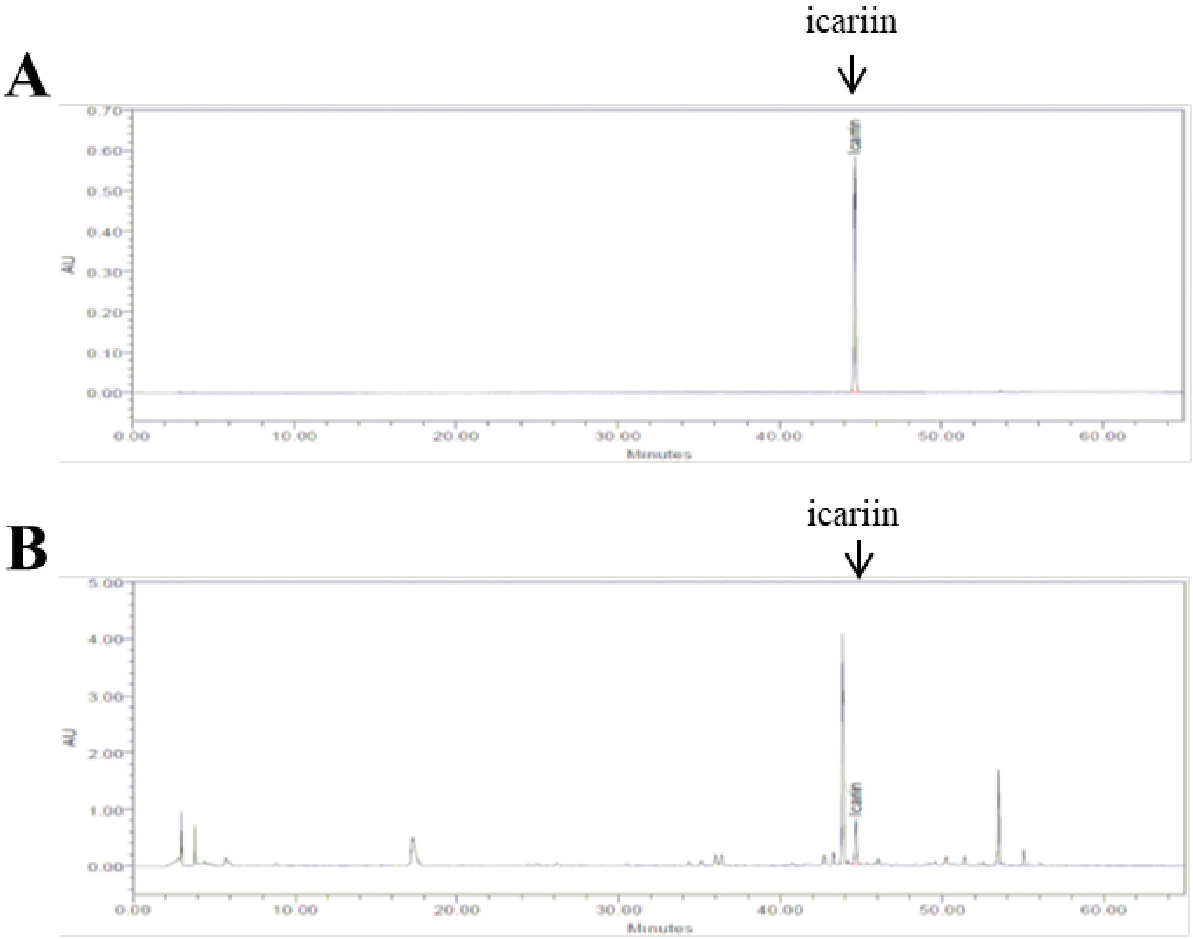


**Supplementary Figure S4. Icariin (ICA) determination by HPLC.**

Represented images showed typical chromatograms for ICA standard solution (A) and *Epimedium* extraction (EE) solution (B).

Unprocessed WB images

Fig. 1G

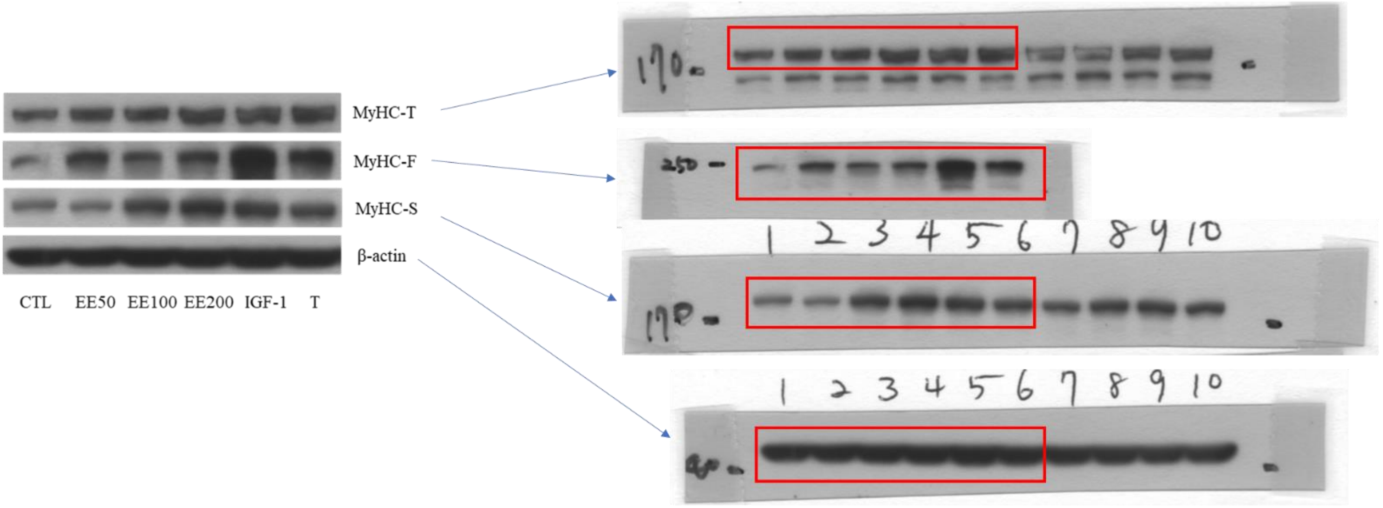


Fig. 2A

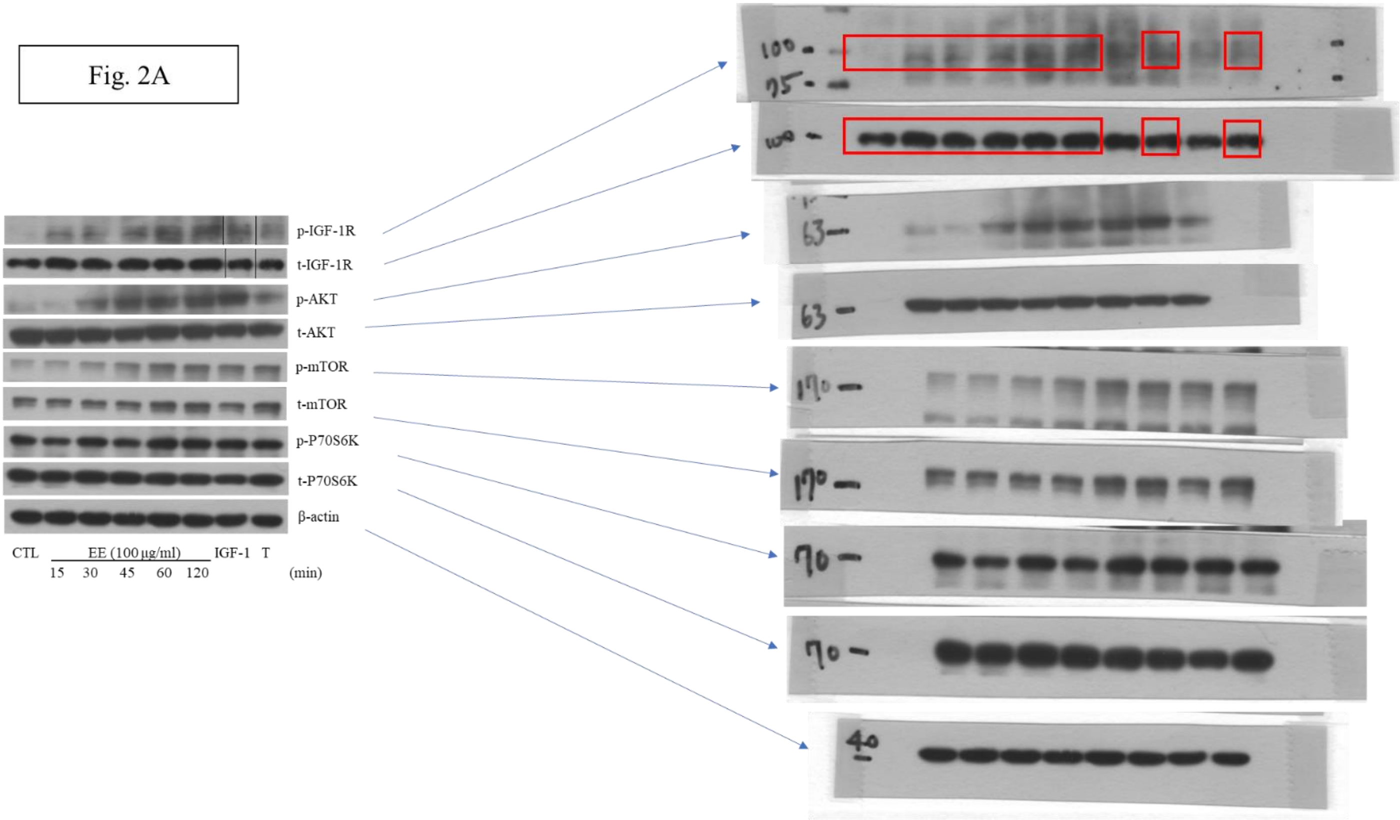

Unprocessed WB images

Fig. 2F

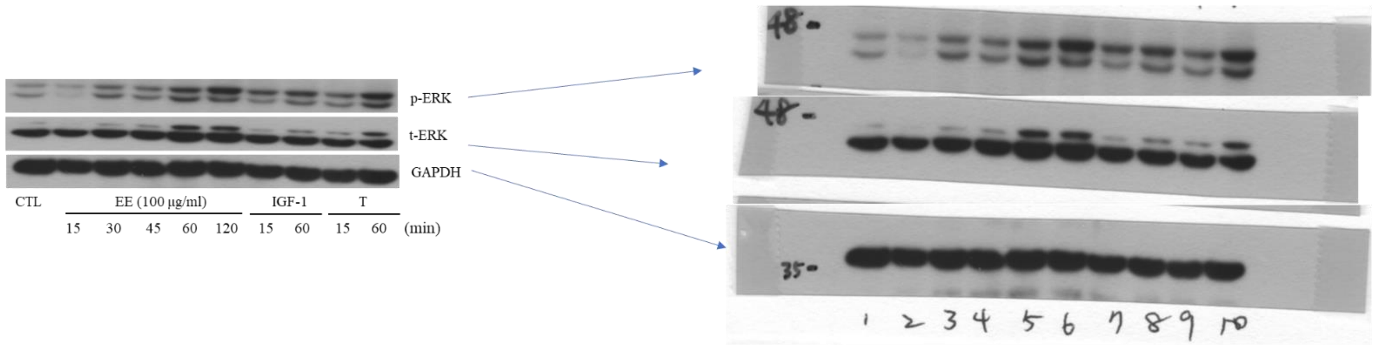


Fig. 2G

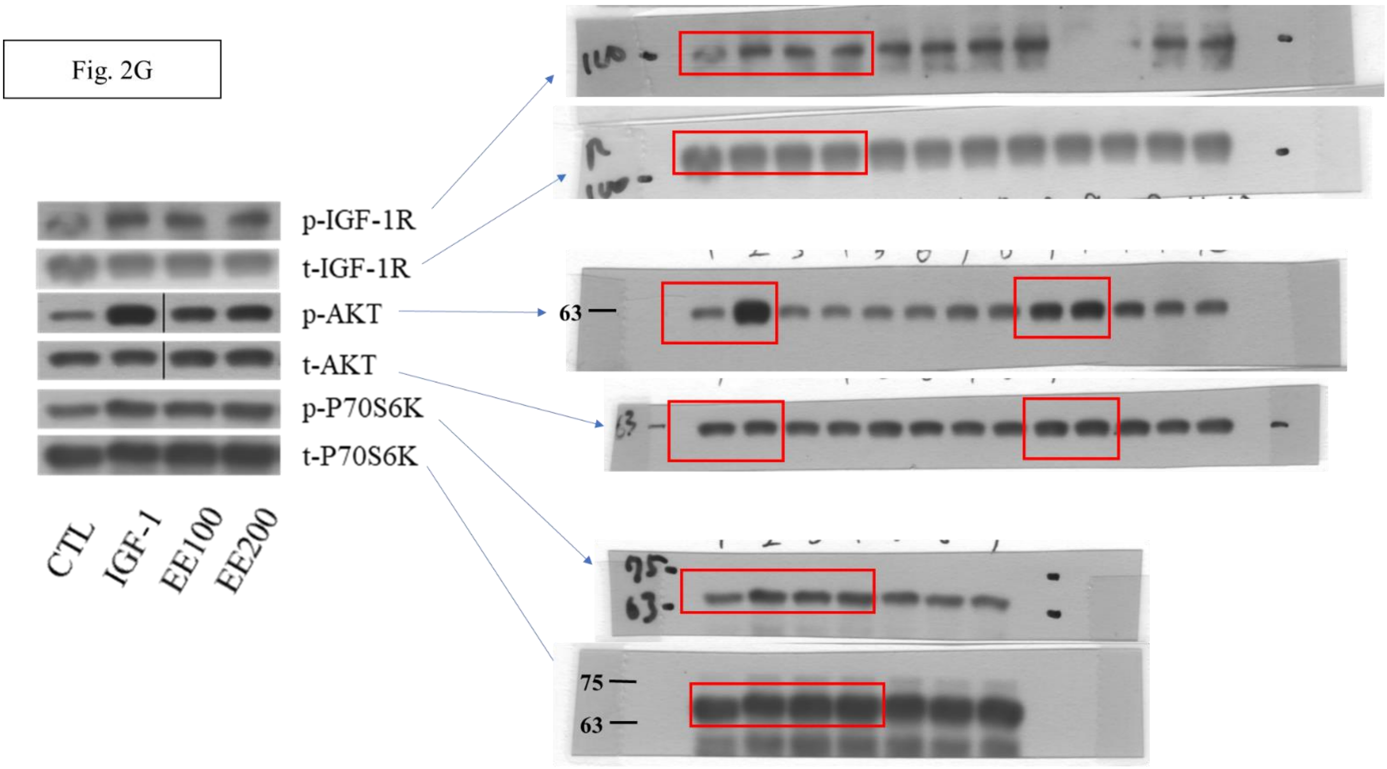


Fig. 2H

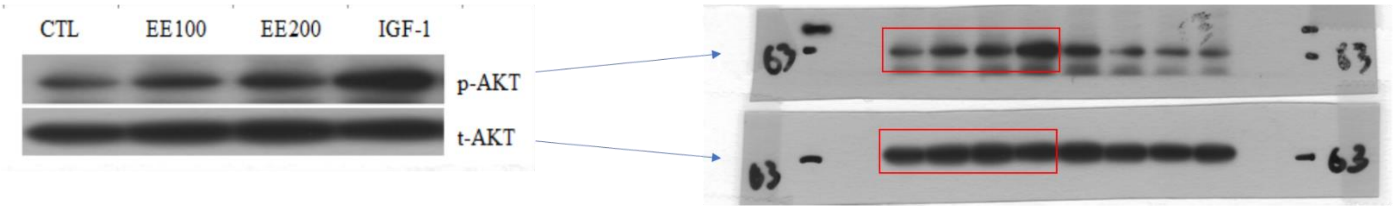

Unprocessed WB images

Fig. 3D

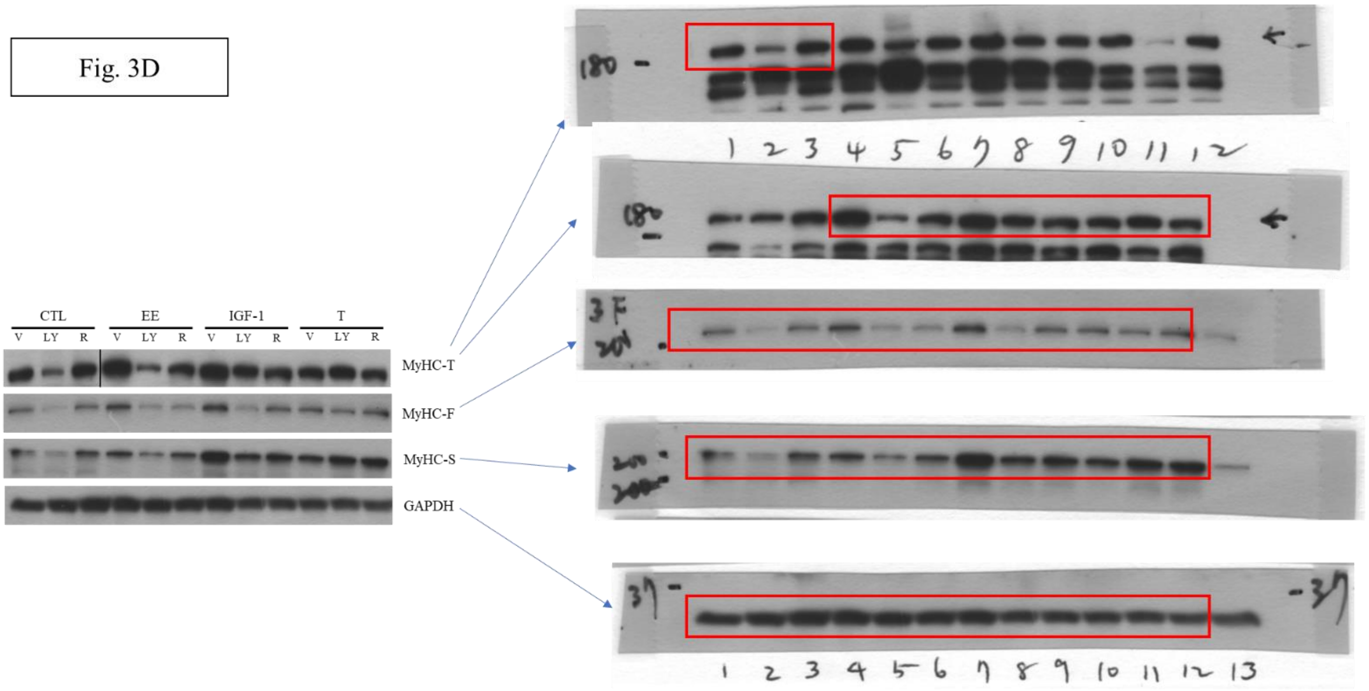


Fig. 3H

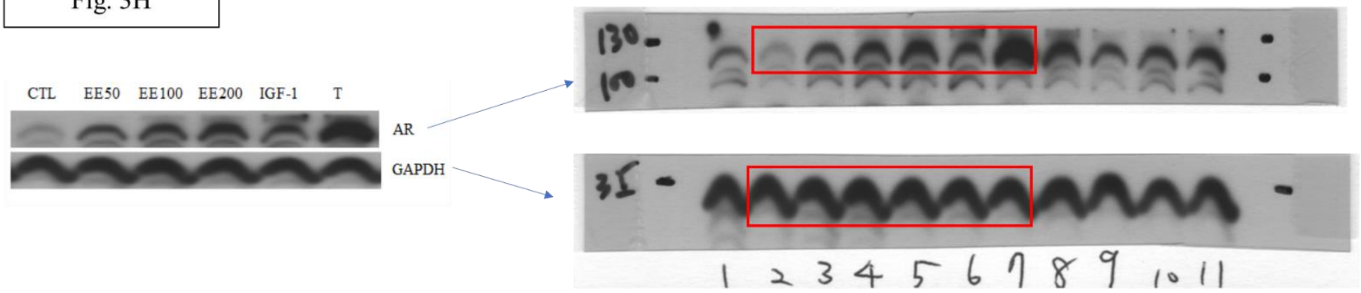


Fig. 3I

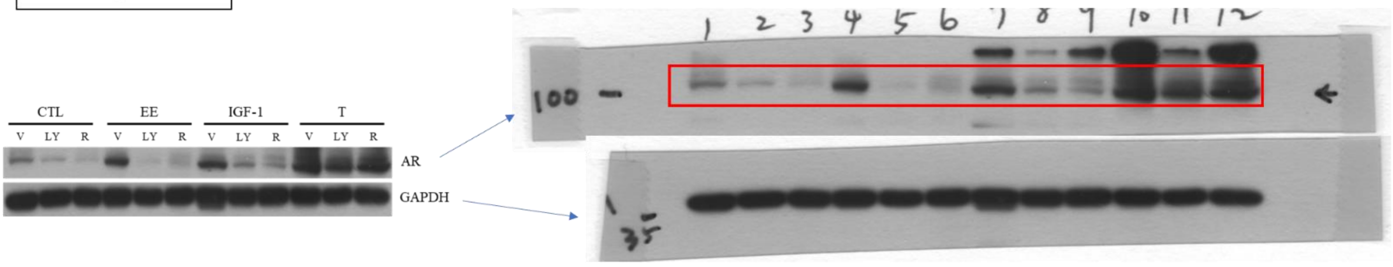

Unprocessed WB images

Fig. 3J

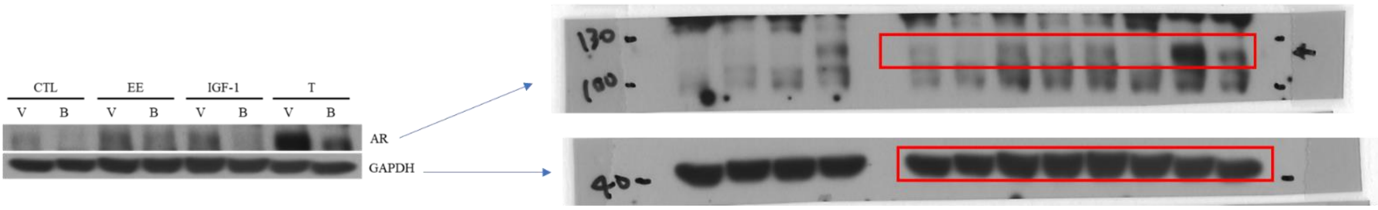


Fig. 3K

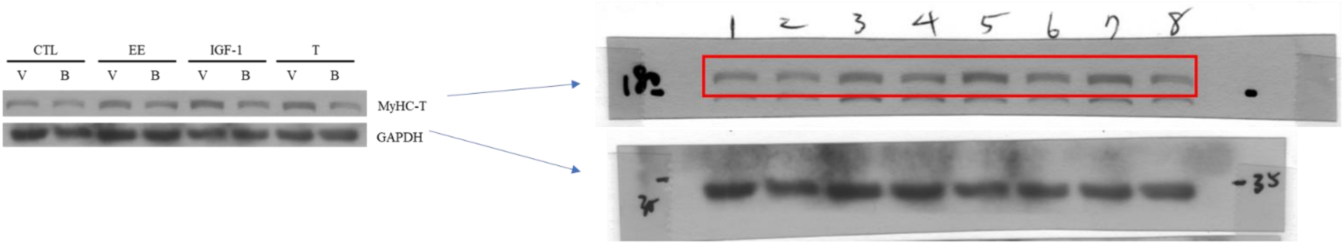


Fig. 4A

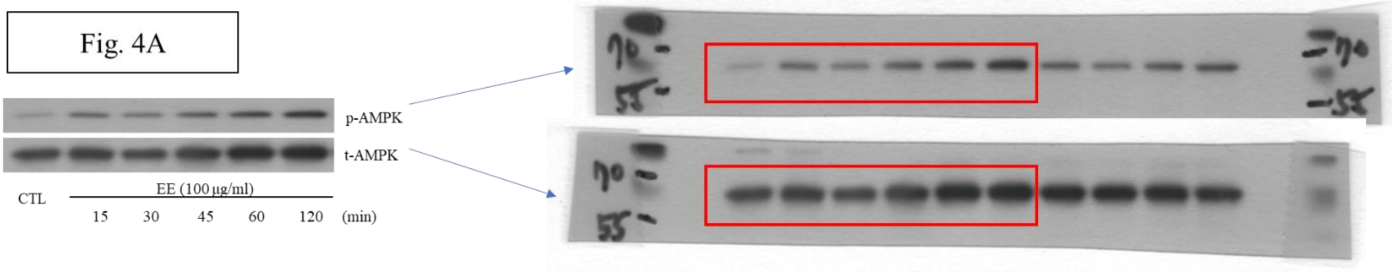


Fig. 4B

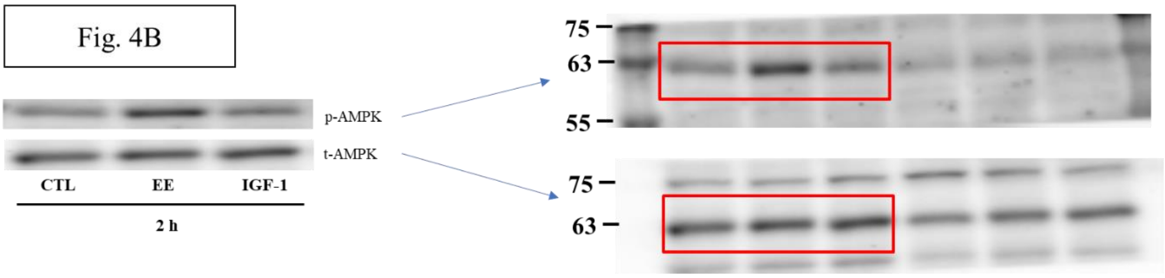


Fig. 4C

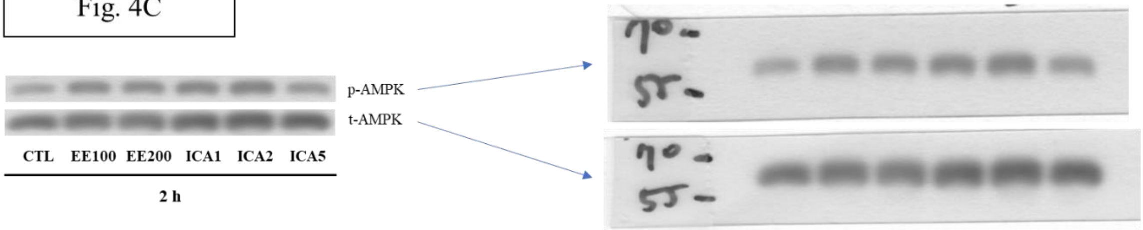

<u>Unprocessed WB images</u>

Fig. 5A

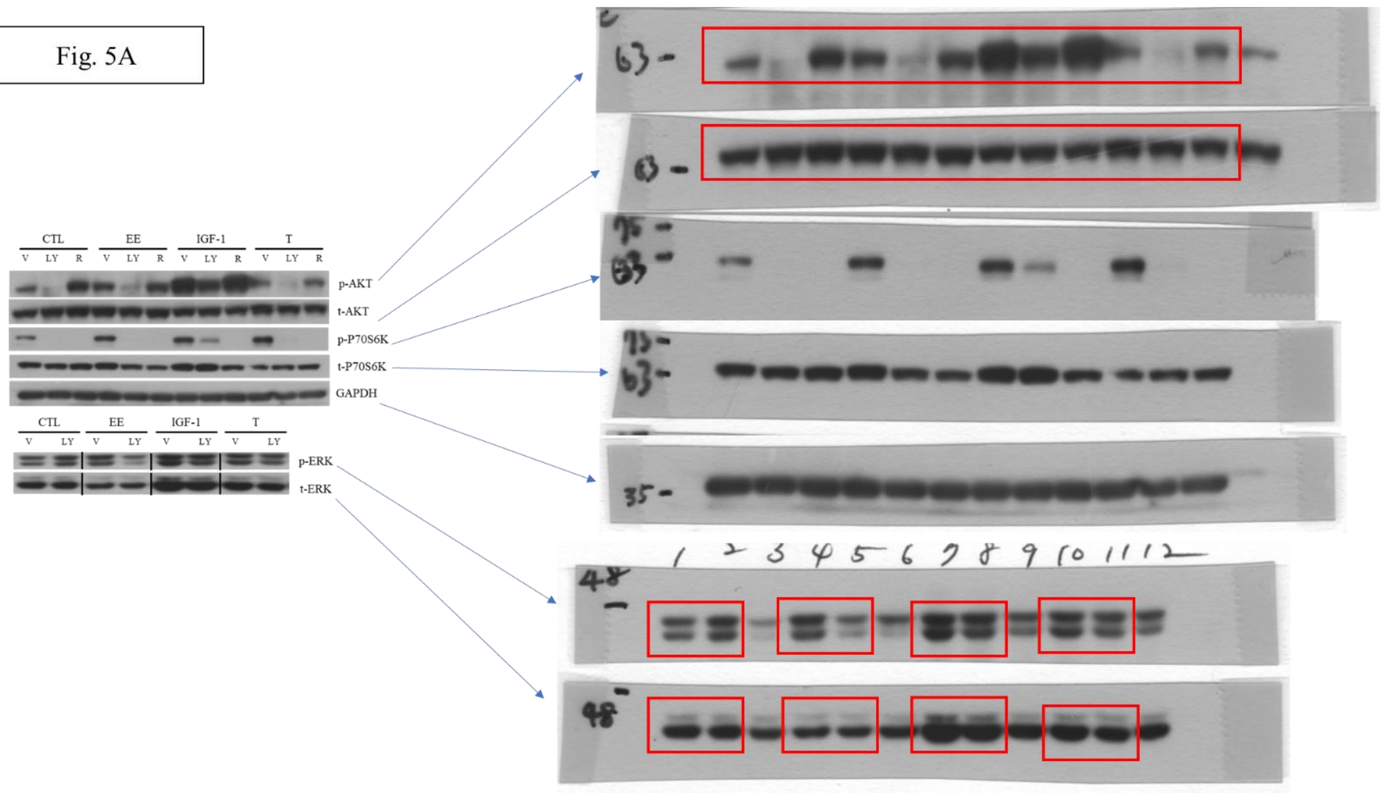


Fig. 5E

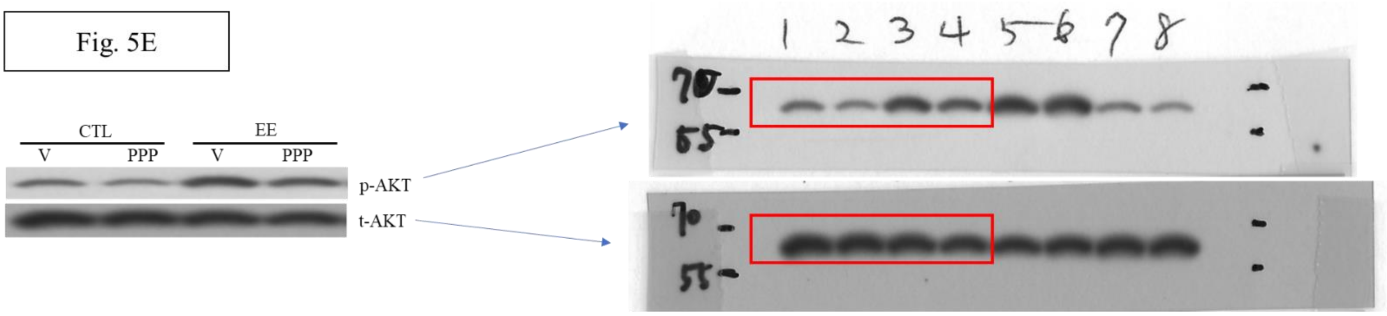


Fig. 5F

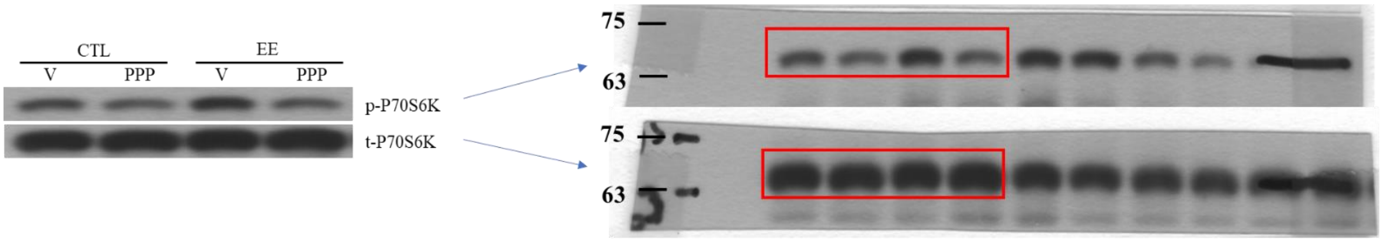

Unprocessed WB images

Fig. 6E

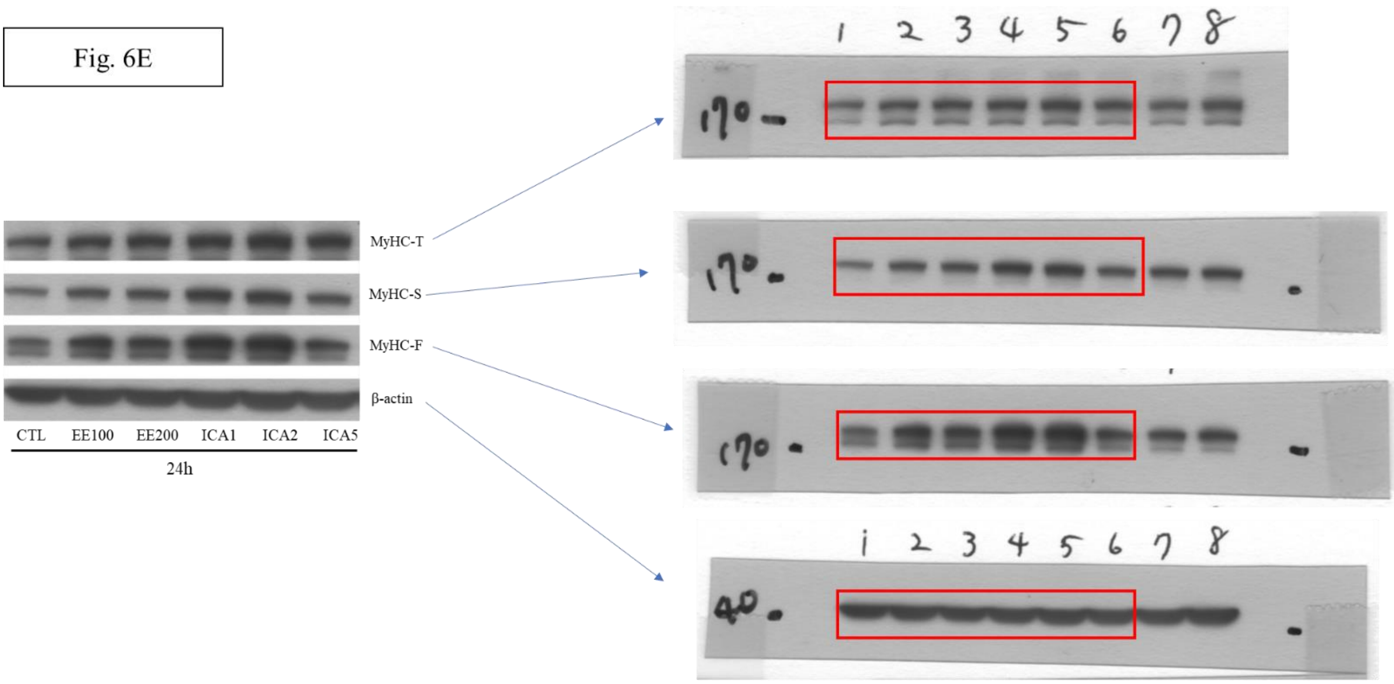


Fig. 6F

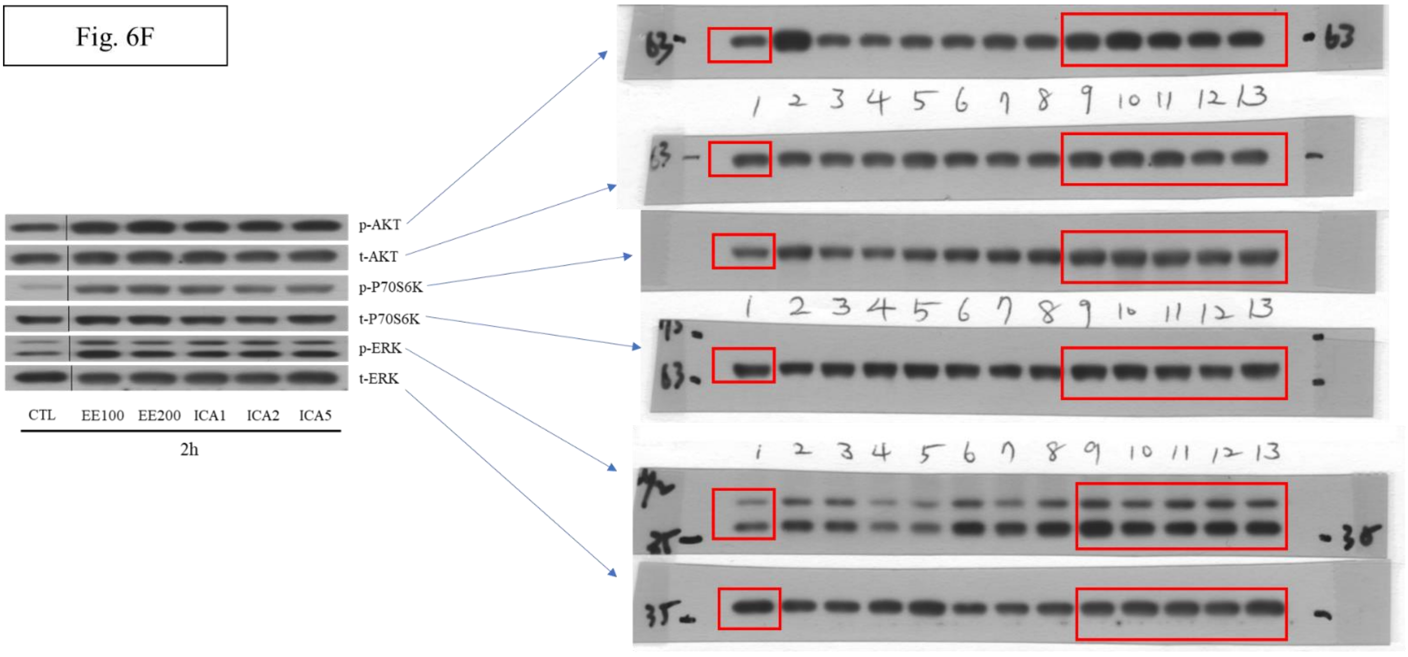

## Unprocessed WB images

Fig. 6G

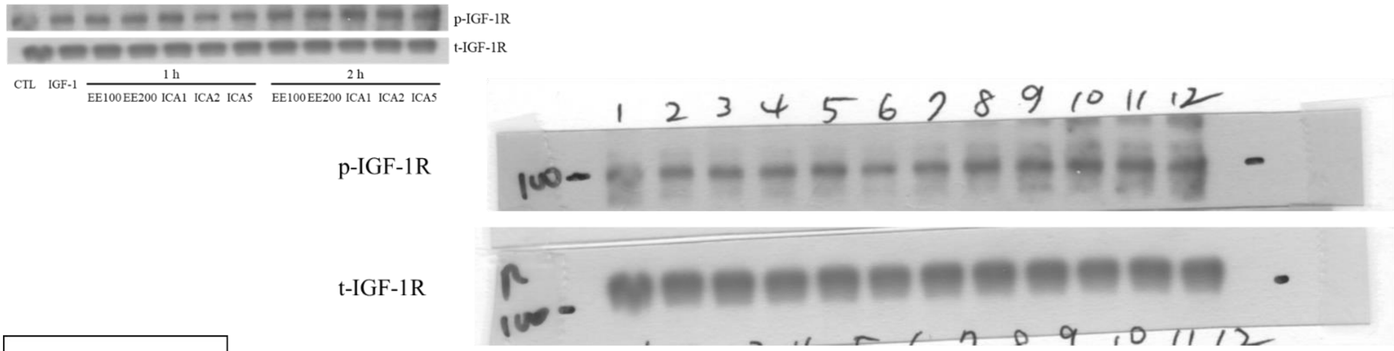


Fig. 6H

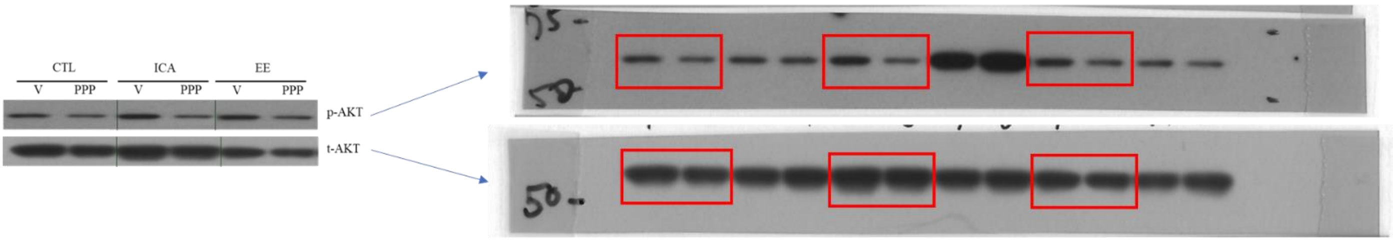


Fig. 7C

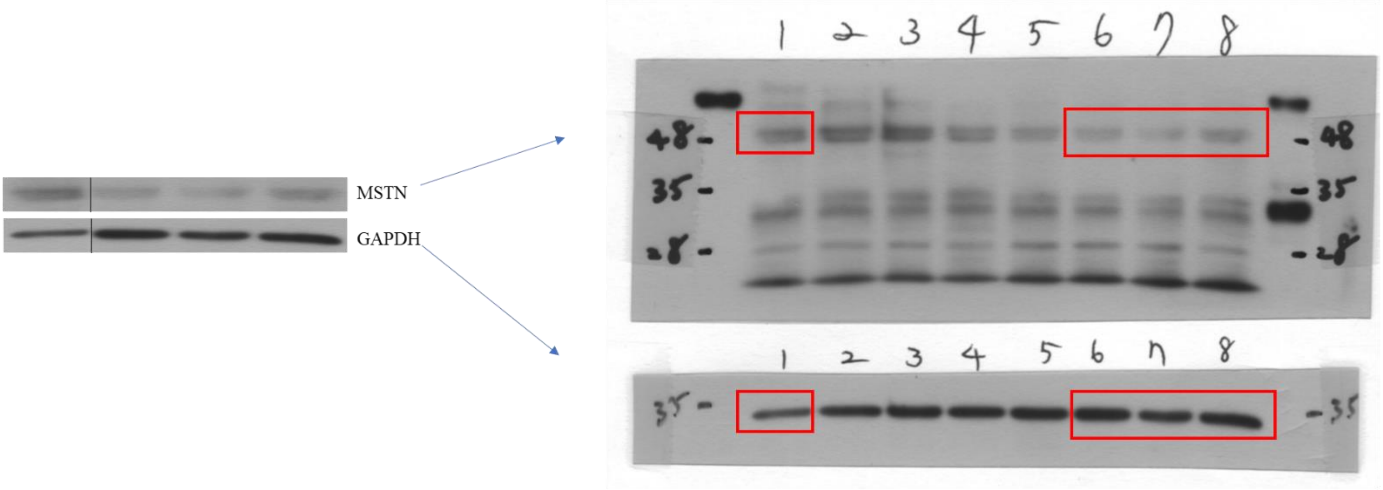

<u>Unprocessed WB images</u>

Supplementary Fig. S1

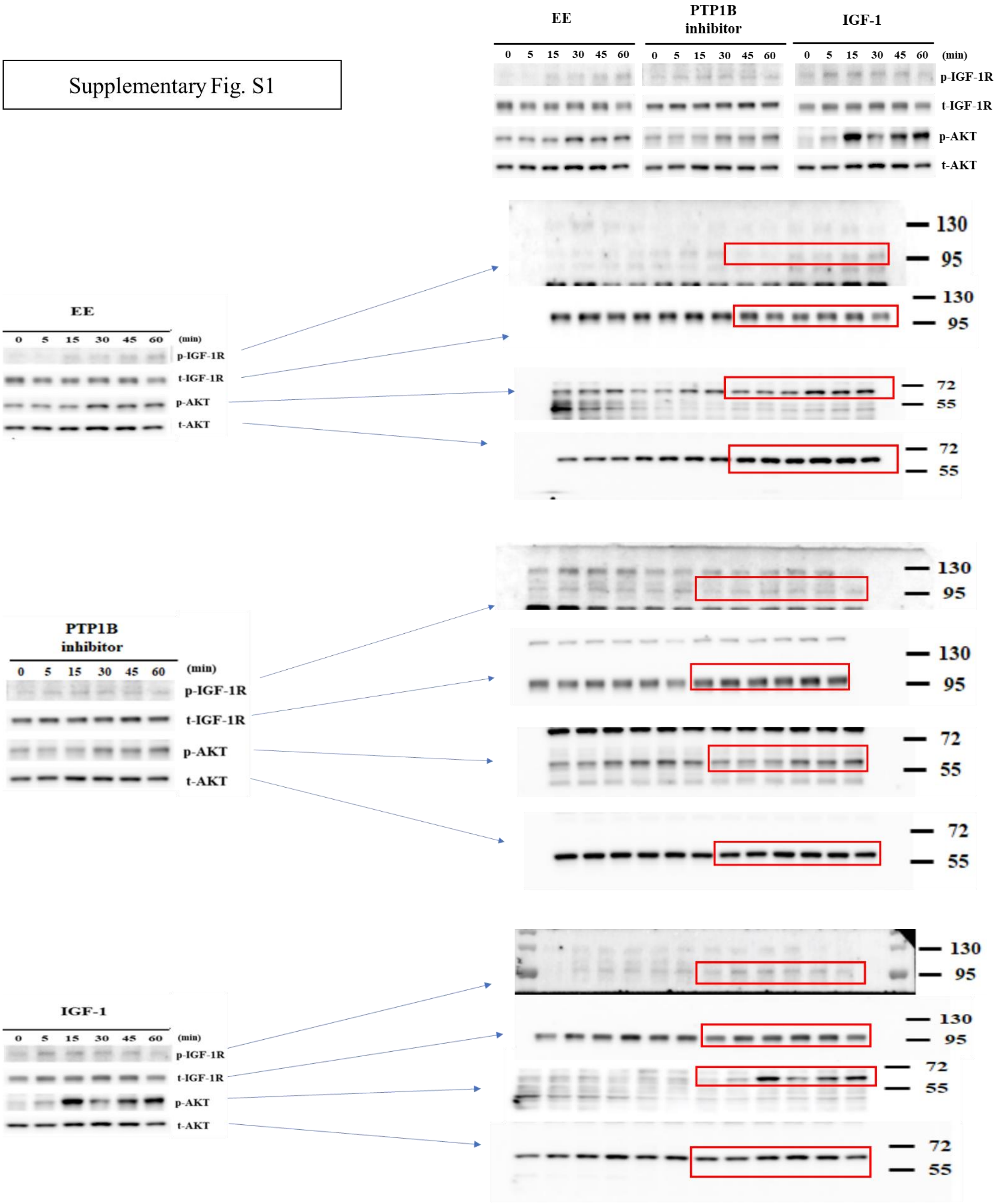

Unprocessed WB images

Supplementary Fig. S2C

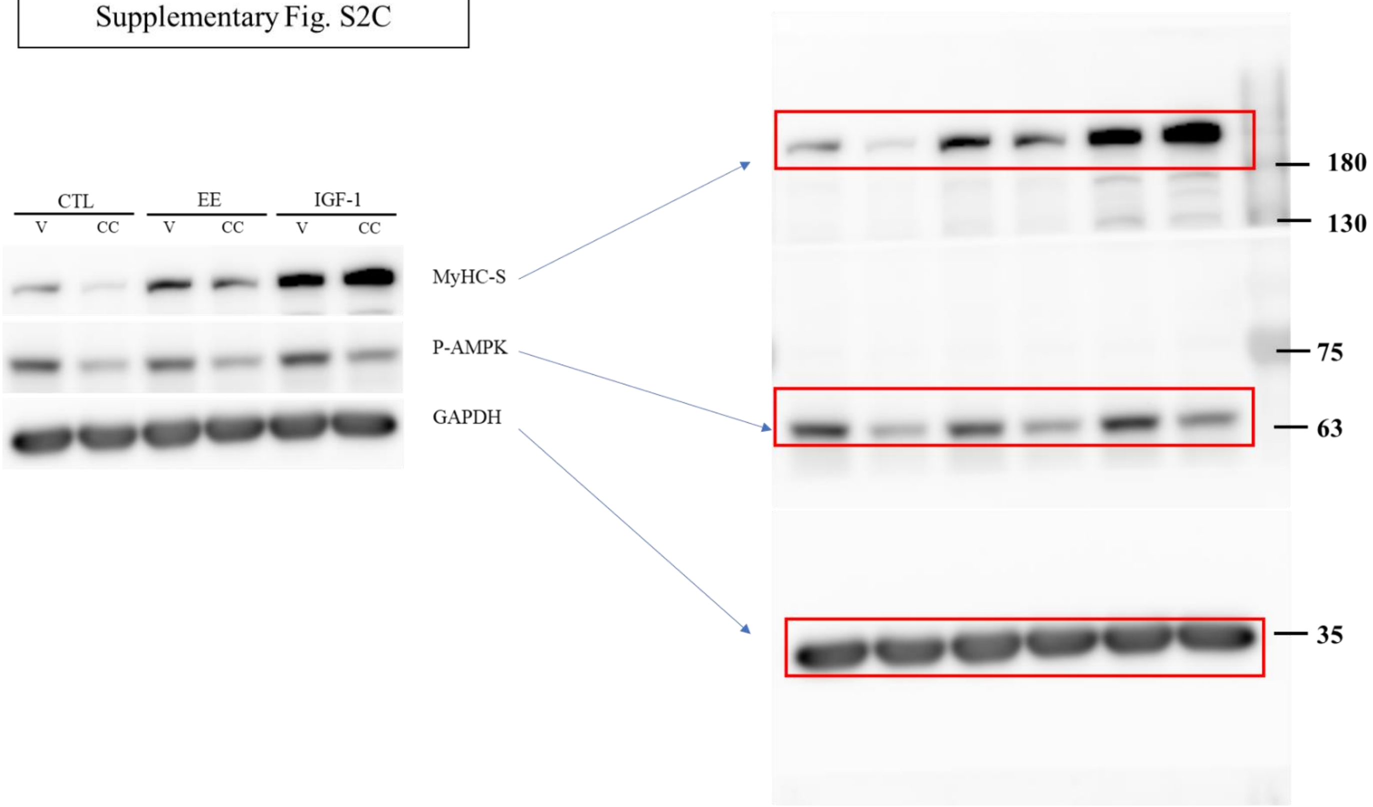

Unprocessed WB images

Supplementary Fig. S3C

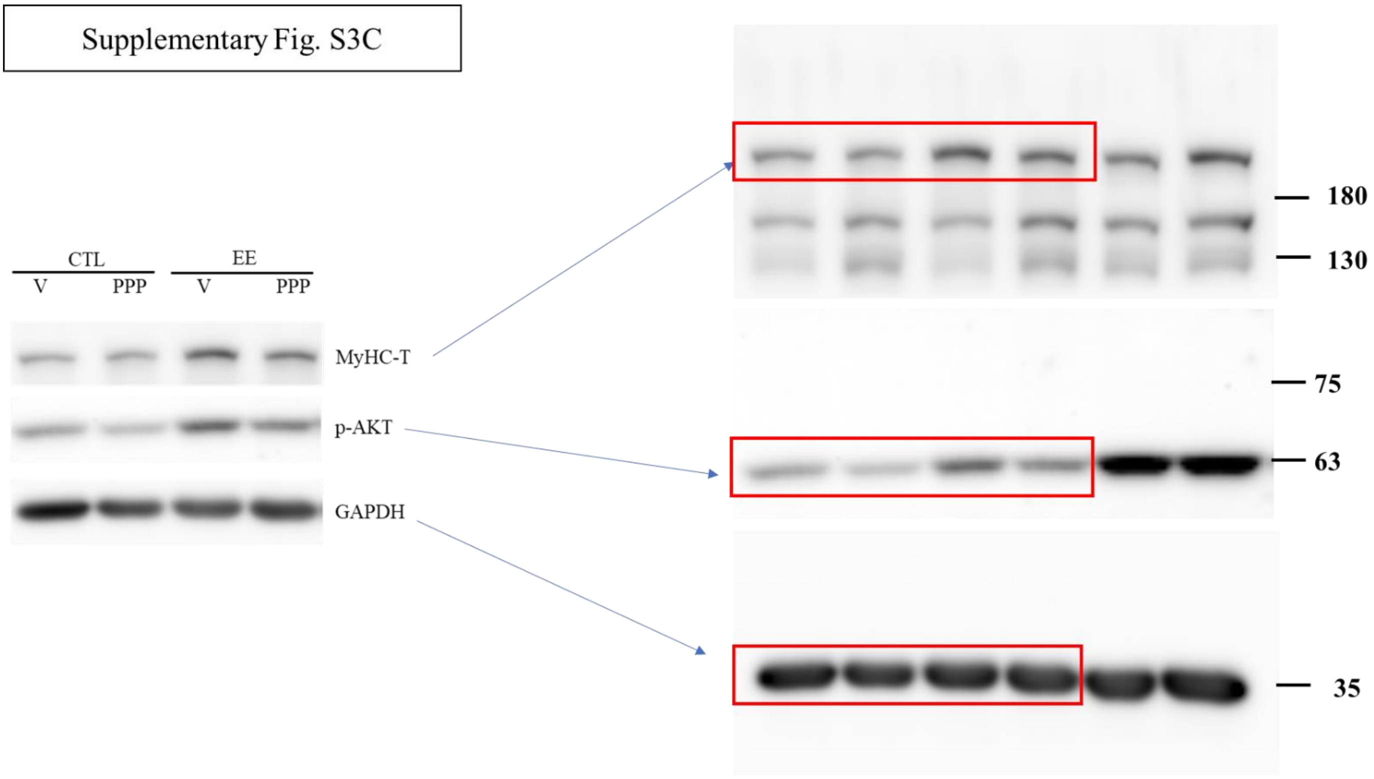

Supplement: Supplementary file 1 — Supplementary Information. [file 41598_2021_89039_MOESM1_ESM.pdf]
